# Striking Phenotypic Variation yet Low Genetic Differentiation in Sympatric Lake Trout (*Salvelinus namaycush*)

**DOI:** 10.1371/journal.pone.0162325

**Published:** 2016-09-28

**Authors:** Kia Marin, Andrew Coon, Robert Carson, Paul V. Debes, Dylan J. Fraser

**Affiliations:** 1 Department of Biology, Concordia University, Montréal, Québec, Canada; 2 Tourism Department, Cree Nation of Mistissini, Québec, Canada; 3 Department of Biology, Concordia University, Montréal, Québec, Canada; 4 Department of Biology, University of Turku, Turku, Finland; 5 Department of Biology, Concordia University, Montréal, Québec, Canada; SOUTHWEST UNIVERSITY, CHINA

## Abstract

The study of population differentiation in the context of ecological speciation is commonly assessed using populations with obvious discreteness. Fewer studies have examined diversifying populations with occasional adaptive variation and minor reproductive isolation, so factors impeding or facilitating the progress of early stage differentiation are less understood. We detected non-random genetic structuring in lake trout (*Salvelinus namaycush*) inhabiting a large, pristine, postglacial lake (Mistassini Lake, Canada), with up to five discernible genetic clusters having distinctions in body shape, size, colouration and head shape. However, genetic differentiation was low (F_ST_ = 0.017) and genetic clustering was largely incongruent between several population- and individual-based clustering approaches. Genotype- and phenotype-environment associations with spatial habitat, depth and fish community structure (competitors and prey) were either inconsistent or weak. Striking morphological variation was often more continuous within than among defined genetic clusters. Low genetic differentiation was a consequence of relatively high contemporary gene flow despite large effective population sizes, not migration-drift disequilibrium. Our results suggest a highly plastic propensity for occupying multiple habitat niches in lake trout and a low cost of morphological plasticity, which may constrain the speed and extent of adaptive divergence. We discuss how factors relating to niche conservatism in this species may also influence how plasticity affects adaptive divergence, even where ample ecological opportunity apparently exists.

## Introduction

Ecological speciation occurs when divergent natural selection in different environments results in reproductive isolation and ultimately new species formation [1]. Its speed and extent across taxa likely depends on multiple factors meriting further investigation [1,2]. Hendry [2] suggested that ecological speciation is best considered as a ‘speciation continuum’, wherein species range from adaptive variation between randomly mating populations all the way to complete and irreversible reproductive isolation between two distinct species. A better understanding of this continuum in evolutionarily young populations of harvested species could improve biodiversity conservation, because evolving phenotypic and genetic differentiation can significantly influence population growth, productivity and species persistence [3,4].

Many north temperate freshwater fishes occupying postglacial lakes, including harvested species, contain phenotypically- and genetically-distinct morphs that have primarily diverged in trophic niche use [2,3,5–7]. They hence provide excellent models for both the study of differentiation along the ecological speciation continuum and its consequences for biodiversity conservation. In several species, such as Arctic charr (*Salvelinus alpinus*) and threespine stickleback (*Gasterosteus* spp.), morphs are at the intermediary stages of ecological speciation. These morphs exhibit varying degrees of genetic differentiation, moderate to relatively high gene flow, adaptive variation to limnetic and benthic feeding niches, and potentially reversible reproductive isolation [2,8–10]. In more extreme cases such as lake whitefish (*Coregonus clupeaformis*), divergent morphs exhibit complete or near complete reproductive isolation, and genetic incompatibilities might arise as a result of divergent selection, or for other reasons due to divergent selection following gene flow reduction [2,11].

Less empirical attention has been directed to diversifying postglacial fishes that exhibit occasional adaptive variation with minor reproductive isolation, and hence to the potential factors influencing the progress or lack of progress at earlier stages of differentiation (but see [10,12]). One species that might typify this situation is lake trout (*Salvelinus namaycush*, Walbaum, 1792), a predator species which exhibits sympatric morphs associated with occupying different habitat niches within several, large North American postglacial lakes [13,14]. The best known morphs are the ‘leans’, ‘siscowets’ and ‘humpers’ of the Laurentian Great Lakes, which occupy mid-depth, deep-water, and drop-off habitats, respectively. The morphs have trophic specializations for consuming different prey [14–16], but appear to exhibit weak genetic differentiation [17]. Morphs in a few other lakes have similarities and differences to Great Lakes morphs. In Great Bear Lake, morphological variation between piscivorous and insectivorous morphs is unrelated to depth [13,18] and up to four weakly, genetically differentiated morphs inhabit shallow waters [19,20]. Conversely, in Atlin Lake, two morphs are not genetically-distinct but have clear depth and habitat preferences [21]. Finally, in Great Slave Lake, three identified morphs display trophic specialization to benthic and pelagic habitats but their genetic differentiation has yet to be studied [14,22]. Overall, previous research suggests that lake trout may be at the earlier-stage end of the ecological speciation continuum in multiple lakes.

**Fig 1:**
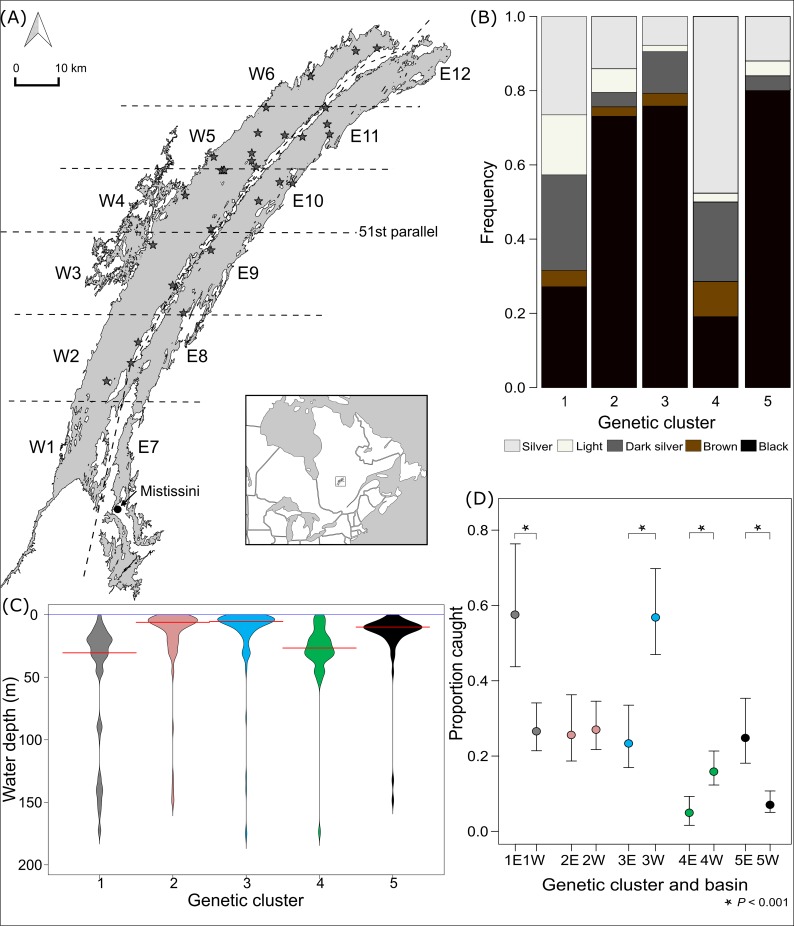
Geographic location of Mistassini Lake, Québec, Canada and colour frequency, depth distribution and basin preferences of each one of the genetically-demarcated clusters. (A) Shown on the map are 24 hr gillnet sets (star) and lake sector (W1–6 and E7–12) divided by dashed lines. (B) Frequency of colours observed in each genetically-demarcated lake trout clusters in Mistassini Lake. (C) Depth distribution of lake trout in each one of the genetically-demarcated clusters. Beanplot shows accurate densities and the red line indicates the median. (D) Basin preferences of lake trout in each one of the genetically-demarcated clusters. Results of a generalized linear model (GLM) that took into account the fishing effort and demonstrates that clusters 1 and 5 were caught in higher numbers in the eastern basin and clusters 3 and 4 were caught in higher numbers in the western.

Mistassini Lake (50.958034°, -73.622340°; Fig 1A) is a large (2,335 km^2^), deep (180 m), postglacial lake in northern Canada [23]. It supports numerous habitat niches that has favoured sympatric differentiation in several harvested fish species, including brook trout (*Salvelinus fontinalis*) and walleye (*Sander vitreus*) [24,25]. For lake trout, Wilson & Hebert [26] suggested that multiple mitochondrial DNA lineages colonized Mistassini Lake after the last deglaciation (7,000–8,000 BP). Two morphs differing in growth, age- and size-at-maturity and depth use were also recently described [27,28]. While these studies offered a glimpse into what such a large and pristine lake might harbour, the small sample sizes, limited spatial distribution and limited genetic assays left an incomplete picture of sympatric differentiation.

We firstly tested and provided evidence that Mistassini Lake harboured multiple genetically- and morphologically-distinguishable clusters of lake trout associated with spatial habitat, depth and fish community structure, through the use of morphological analyses, catch locations, bycatch data and microsatellite DNA surveys. Yet genetic differentiation was low and genetic clustering was largely incongruent among several population- and individual-based clustering approaches. Moreover, genotype- and phenotype-environment associations were either inconsistent or weak. We thus also evaluated genetic differentiation in relation to effective population sizes and the extent (and possible asymmetry) of contemporary gene flow, and considered additional ecological factors within Mistassini and other large postglacial lakes that might inhibit genetic differentiation in lake trout and other postglacial fishes. Our work contributes to a better understanding of how intraspecific phenotypic plasticity might affect the trajectory and progress towards ecological speciation.

## Materials and Methods

### Fish sampling

Between June 12 and July 29, 2013, 636 lake trout were sampled throughout Mistassini Lake using monofilament nylon gillnets and angling, in order to obtain genetic (n = 636) and morphological data (n = 281). Gillnet design was based on previous lake trout research in large lakes (183 m long, 1.8 m tall; six 30.5 m gangs, one for each mesh size, ranging 51–114 mm stretch mesh; [27], and permitted unbiased targeting of all known sizes of Mistassini lake trout [29]. Twenty-nine gillnets were set throughout the lake (Fig 1A) in areas known by local fishers to have lake trout, from depths between 3–178 m, were soaked overnight and lifted after a 24-hour period. Gillnets were lifted slowly, bycatch were recorded and returned to the lake immediately, and trout were placed in fresh water baths with aerators. All living trout were anaesthetized with buffered tricaine methanesulfonate (MS-222) and subsequently processed. From each trout, we collected and preserved a small piece of adipose fin tissue in 95% ethanol until DNA extraction, took a standardized photograph (see below), measured total and fork length (TL and FL, respectively, ± 1 mm) and wet mass (± 50 g), and recorded net or lure depth and location of capture (GPS). Tissue samples were also donated by local anglers. To account for relative spatial location when GPS data were unavailable for angled samples, the location was reported as a sector, pre-determined on a map provided in a sampling kit. Each basin was separated into six sectors (Fig 1A). Research undertaken in this study complies with the requirements of the Canadian Council on Animal Care (CCAC). This protocol was approved by Concordia University Office of Research–Research Ethics and Compliance Unit (permit number: 30003281) and the Ministère des Ressources naturelles direction de l’expertise Énergie-Faune-Forêts-Mines-Territoire du Nord-du-Québec (permit number: 2013-04-03-107-10-S-P).

### Genotyping

We extracted and analyzed DNA from 636 trout by amplifying 19 microsatellite loci using multiplexed polymerase chain reactions (PCRs; see S1 File for loci and PCR details). Amplified products were electrophoretically migrated and allele sizes scored using a 3500x Genetic Analyzer, associated size standards and software (Applied Biosystems Inc.). To estimate repeatability, 16 samples were independently genotyped and scored three times.

### Genetic diversity

We tested for the presence of null alleles, large allelic size dropout, or scoring errors associated with allelic stuttering issues in our dataset using MICROCHECKER (v.2.2.3) [30]. Descriptive genetic statistics, including number of alleles (N_A_), expected (H_E_) and observed (H_O_) heterozygosity for each locus were computed using FSTAT (v.2.9.3.2.) [31]. To ensure that loci fulfilled assumptions of selective neutrality in Hardy-Weinberg equilibrium (HWE) tests and in clustering analyses below, tests of selection were performed using ARLEQUIN (v.3.5.1.2) [32]. We then tested for deviations from HWE and linkage disequilibrium (LD) using GENEPOP (v.4.2) [33] (i) under the null hypothesis of one randomly mating population within Mistassini Lake, but also (ii) under the alternative hypothesis that genetic structuring was present, defined as multiple, individual based clusters inferred from the Bayesian model-based clustering software STRUCTURE (v.2.3.4) [34]. If the null hypothesis was rejected, we expected many more HWE deviations to exist when assessing the lake as one cluster vs. multiple ones.

### Genetic clustering/differentiation

#### Non-spatial clustering

We employed both non-spatial and spatially informed (see below) approaches to characterize genetic clustering and structure. Non-spatial analyses were performed using three different methodologies (see S2 File); however, we only report the methods and results from STRUCTURE in the main paper, as the most commonly used clustering software with more easily biologically-interpretable results. Namely, this software consistently defines clusters (K) [35] and accounts for admixture [34], which is important for situations of low F_ST_ and multiple glacial lineages. We ran STRUCTURE with no *a priori* information under models of K = 1–20, a burn-in period of 500,000 followed by 2,000,000 iterations, and replicated 20 times each per K. We estimated the most likely K value based on the *ad hoc* ΔK statistic of Evanno et al. [35] as implemented in STRUCTURE HARVESTER [36] and the highest mean value of the log-likelihood of the data. The 20 iterations per K value were then combined into a single output, providing an overall inferred ancestry coefficient (*q*) for each individual using the program CLUMPP (v.1.1.2) [37]. Because STRUCTURE can over estimate K when closely related individuals are present in a sample [38] we estimated the number of full siblings using COLONY (v.2.0.6.1) [39] as a supplementary analysis, assuming male and female polygamy and default parameter settings over three runs. We then randomly removed one half of each full sibling pair and reran STRUCTURE with the same parameter settings as described above. COLONY results revealed a low number of full sibling pairs (8, i.e., 16 of 636 individuals) from separate families and their exclusion did not affect the mean LnP[D] output from STRUCTURE (data not shown). Therefore, all subsequent analyses were based on the full dataset. The extent of genetic differentiation (F_ST_) [40] between defined clusters was quantified using GENEPOP. We also calculated pairwise F_ST_ for both body and head morphological clusters (see below) to determine if genetic differentiation was higher among morphological vs. genetic clustering.

Genetic differentiation may be influenced by stepwise mutations that are accounted for in measuring R_ST_ rather than F_ST_, which in young postglacial lakes can reflect population divergence predating lake age [41]. We assessed and confirmed that R_ST_ > F_ST_ in Mistassini Lake (S3 File). Nonetheless, to make our study comparable to previous lake trout research, subsequent analyses were based on F_ST_ only, because F_ST_ (i) typically has lower standard errors than R_ST_ [42], (ii) is more precise when high gene flow is detected [43], and (iii) performs better than R_ST_ for most typical sample sizes [44].

#### Spatial differentiation

To complement non-spatial clustering analyses and provide insight into individual-level genetic relationships, we tested for associations between geography (longitude, latitude and depth) and the distribution of individual genetic variation across the lake. The advantage of these spatial analyses is that they do not rely on HWE or linkage equilibrium and thus are useful for detecting gradients and complex spatial-genetic structures [45]. We were able to obtain GPS (± 5 m) and depth (± 1.8 m) on 554 of 636 trout for these analyses. Similarly to above, we performed spatially-informed analyses using three different methods (see S2 File); however, we only report the methods and results from a spatial principal components analysis (sPCA) performed using the ‘adegenet’ package (v.1.3–1) [46] in R [47]. An sPCA compares the allelic frequency of individuals to allelic frequencies of their neighbours using Moran’s index (*I*) [45,48]. Although our samples were collected relatively evenly across the lake, some were more heavily sampled and so we used two different connection networks to define which individuals were neighbours in the sPCA algorithm: (i) Delaunay triangulation [49], which is suited for uniform sampling and commonly used in a number of different spatial programs [50]; and (ii) inverse distances, which is recommended when sampling is aggregated [45] and assumes all individuals are neighbours. We tested for significant global (i.e., neighbouring individuals are more similar than expected) and local (i.e., neighbouring individuals are more dissimilar than expected) structures using 999 permutations.

### Contemporary effective population sizes and gene flow

We estimated the contemporary effective population size (*N*_e_) for each defined genetic cluster using the linkage disequilibrium method implemented in LDNe (v. 1.31) [51]. Lake trout were not aged, so our cluster samples consisted of overlapping generations which downwardly biases *N*_e_ estimates up to 25–30% [52]. We estimated contemporary gene flow (recent migration rates, *m*, within the last few generations) between cluster pairs using all individuals with BayesAss (v.3.0) [53]. Five separate iterations were performed using 10^7^ Markov chain Monte Carlo (MCMC) iterations with a burn-in period of 10^6^ and sampled at a frequency of 100. Prior to each run, mixing parameters (allele frequencies, inbreeding and migration rates) were optimized until acceptance rates were between 20–35%, as recommended in the manual. To assess whether MCMC had converged successfully for each run and overall, we used the program TRACER (v.1.6.0) [54]. Whether mutation-drift equilibrium conditions had been reached among defined genetic clusters was assessed using Whitlock’s [55] equation for the time, in generations, required for F_ST_ to reach halfway to a new equilibrium:

$t\frac{1}{2}=\ln(\frac{1}{2})/ln[{\left(1-m\right)}^{2}{\left(1-2Ne\right)}^{-1})]$, where *m* = mean gene flow into each cluster.

### Morphological analysis

#### Body and Head Morphology

To characterize body morphological variation, we took a full-bodied standardized photograph of each trout immediately after capture using a digital camera (Nikon D3100) with a UV filter mounted on a tripod. Many tissue samples were donated from local fishers, so we were only able to take photos of a total of 281 of 636 trout. We placed each fish on its right side on a flat piece of plywood with the dorsal, caudal and anal fins in open positions; if fish were bent or distorted, photographs were discarded. Eighteen landmarks (Fig S4.1A in S4 File) were then digitized on each trout using tpsDig2 (v.2.17) [56]. A separate morphological analysis was performed on the head with three landmarks and 22 semi-landmarks used to measure curvature, following Zimmerman et al. [57] (Fig S4.1B in S4 File). Ten equally-spaced regions were produced by a reference grid and superimposed on each photograph between the snout and opercle using MakeFan (v.8) [58]. Semi-landmarks were then slid along both upper and lower curves using tpsDig2, aligning semi-landmarks perpendicularly to the curve by reducing the bending energy among individual points [59]. Landmark and semi-landmark positions utilized are common in studies of lake trout and related species, and were selected to measure traits commonly associated with adaptation in swimming and foraging performance [8,24,57,60].

Geometric morphometric analyses were conducted separately on body and head morphology datasets using tpsRelw (v.1.36) [61]. TpsRelw takes into account spatial variation among assigned landmarks relative to all others using a thin-spline analysis [62]. It specifically produces partial warps, which are geometric constructs derived from the amount and direction of bending required to change the consensus shape [59]; principal components of these partial warps scores are termed relative warps (RWs), which quantify body shape and head variation [63]. The first seven RWs for body shape (78% of variation) and first three RWs for head shape (70% of variation) were used for subsequent statistical analyses. We identified morphological clusters with MCLUST (v.4.4) [64] as implemented in R. Tested models assumed that the data represented one to up to 10 clusters. All models were analyzed using two (EII, VII) of the nine multivariate mixture options; the model with the highest Bayesian information criteria (BIC) was selected as best describing morphological clusters [65]. To measure the reliability of classification of individual fish to MCLUST cluster, we performed body and head discriminant function analyses (DFA) with jackknifing in PAST (v.3.08) [66].

#### Body size and colouration

Body size (mass) and the relationship between mass and total length (TL) was compared among genetic clusters using linear models. In the latter model, the natural logarithm (ln) of mass was regressed onto the ln of TL, genetic cluster and their interaction. A subsequent pairwise comparison of the inferred slopes (mass/length curves) was conducted using ‘lsmeans’ package (v.2.17) [67]. Mass was recorded for 520 and TL for 472 (of 636) trout.

Colour frequency distributions were compared using a χ^2^ test in the ‘vcd’ package (v.1.4–1) [68]. The colour of 422 (of 636) trout was classified and recorded at the time of capture based on being black, brown, dark silver, silver or light (i.e., white, light silver).

### Associations between genetic, morphological and ecological differentiation

#### Genetic & morphological or ecological associations

We tested for an association between genetic and morphological clusters using a contingency test. We then assessed whether the spatial distribution of genetic clusters was influenced by geography (lake basin, sector within basin) and/or by water depth, using a redundancy analysis (RDA) within the ‘vegan’ package (v.2.3–1) [69], which incorporated raw *q* values for each individual. We used a global permutation test of the RDA result to test for a relationship between genetic cluster and ecological variables, as well as a permutation test on canonical axes with 1,000 steps [70]; R^2^ values were calculated as a measure of ‘goodness-of-fit’. The RDA was complemented with a similar multinomial regression analysis which, in contrast, incorporated all individuals weighted based on their highest *q* value. Because the RDA and multinomial regression analyses yielded largely congruent results, only results for the former are reported in the paper (see S5 File for multinomial regression details). Differences in the spatial distributions of clusters among basins were also assessed using a generalized linear model (GLM) fitted with a binomial error distribution and logit link-function (proportion data).

#### Genetic & fish community structure associations

Based on recorded gillnet bycatch data, we tested whether genetic clusters were associated with fish community structure using a contingency test. Bycatch abundance and diversity captured within the same net/panel as each trout provided a reasonable proxy for prey availability and the extent of interspecific competitor species. The relationship between genetic clustering and fish community structure was visualized using a RDA.

#### Mantel tests: genetic differentiation vs. morphological or ecological differentiation

We further tested for associations between the extent of genetic differentiation among defined clusters and either morphological differentiation, depth, fish community structure (abundance), or colour dissimilarity, by using Mantel tests implemented in the ‘vegan’ R package. Mantel tests always compared two pairwise distance matrices: cluster pairwise F_ST_ values and either the absolute difference in (i) mean scores for the first seven RWs for body morphology and first three RWs for head morphology, (ii) median depth, (iii) prey abundance, or in (iv) colour frequencies.

### Statistical notes

Unless otherwise stated, we corrected multiple comparison *P*-values to adjust for any potential type I errors following the false discovery rate procedure [71]; significance was defined at the nominal alpha value of 0.05.

## Results

### Genetic diversity

A high repeatability of genotyping (100%) was confirmed for the 16 samples independently run and scored three times. The 19 loci averaged 29.5 alleles per locus (range of 13–72) with an average H_O_ across loci of 0.813 (range of 0.526–0.939; Table S6.1 in S6 File). No locus showed evidence of being under selection, nor was there any evidence of scoring errors as reported from MICROCHECKER. Under the null hypothesis that all 636 trout were from one randomly-mating population, heterozygote deficiencies were detected at 15 of 19 loci (*P* < 0.050; Table S6.2 in S6 File). These deficiencies were greatly reduced at the level of clusters defined by STRUCTURE, providing a first indication of genetic structuring (28 of 95 cluster-locus comparisons at uncorrected *P*-values (0.000–0.049); the remaining 17 deviations from HWE (*P* = 0.000–0.044) were spread across all five clusters and across nine loci. We also detected significant LD in only seven of 171 locus-by-cluster tests (20 of 171 tests at uncorrected *P*-values), and found little evidence for LD when accounting for clusters (12 of 855 tests; Table S6.3 in S6 File). Significant LD tests were spread across all clusters and among 23 unique loci-pairs.

### Genetic clustering/differentiation

#### Non-spatial clustering

Results from STRUCTURE and STRUCTURE HARVESTER suggested five genetic clusters in the lake (ΔK = 57.03 mean LnP[D] = -58060.42; Table S6.4 in S6 File), with a global F_ST_ of 0.017 and significantly greater than zero. Pairwise estimates of F_ST_ between clusters ranged from 0.012 to 0.036 (Table S6.5 in S6 File); cluster 2 vs. 3 and 4 vs. 5 exhibited the lowest and highest levels of differentiation, respectively. Pairwise estimates of F_ST_ between morphological clusters (see below) were lower and ranged from 0 to 0.002 (body; Table S6.5 in S6 File) or 0.003 (head)).

#### Spatial differentiation

**Fig 2:**
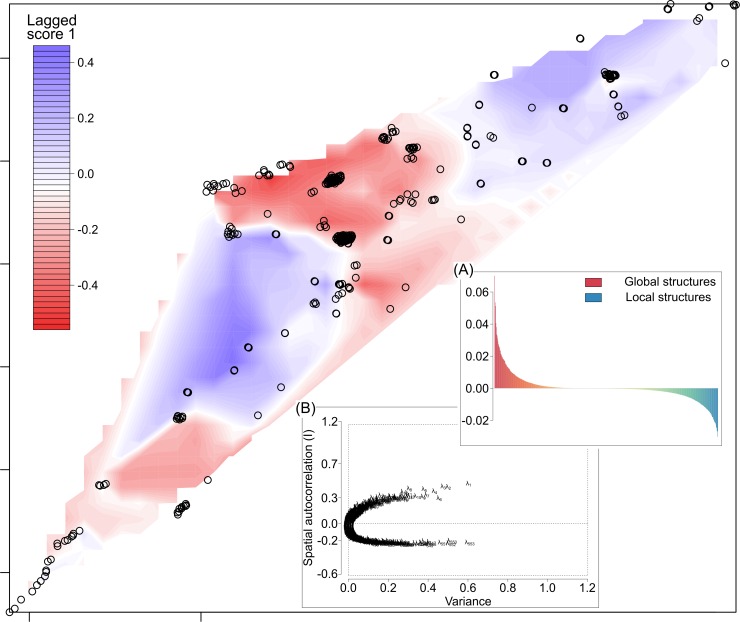
Spatial principal component analysis of lake trout showing the first global structure across Mistassini Lake. Plotted are the lagged scores in which colours (blue to red) represent the score of an individual genotype; each is positioned by its spatial coordinates. Inset is the barplot for all eigenvalues (A), and the screen plot (B) which illustrates the spatial and variance components of those eigenvalues.

The global permutation test on the eigenvalues derived from the sPCA using a Delaunay connection network revealed significant global structure (nper = 999, *P* = 0.028) but no significant local structure (nper = 999; *P* = 0.621). Screen- and bar-plots of the eigenvalues (Fig 2) suggested that only the first global axis should be retained. This axis revealed genetic complexes in which individuals were more closely related than expected, with particular nuances existing in the western basin near the mouth of the Rupert River and in the north near the Big Pass (Fig 2). Using the inverse distance connection method did not significantly change the results (not shown).

### Contemporary effective population sizes and gene flow

Point estimates of *N*_e_ in general had fairly tight confidence intervals and ranged from 371 (cluster 5) to 4,199 (cluster 2; Table S7.1 in S7 File). Estimates of *m* were quite high, ranging from 0.002 to 0.091, with a global average of 0.017 (Table S7.2 in S7 File). Asymmetries in *m* existed, namely higher *m* was detected from cluster 1 to clusters 2, 4 and 5 than vise-versa (Fig S7.1 in S7 File). Generation times (t) for F_ST_ to reach a new equilibrium between cluster-pairs ranged from 22 (clusters 2–5) to 177 generations (clusters 1–3), suggesting migration-drift equilibrium has been reached.

### Morphological Analysis

#### Body and Head morphology

**Fig 3:**
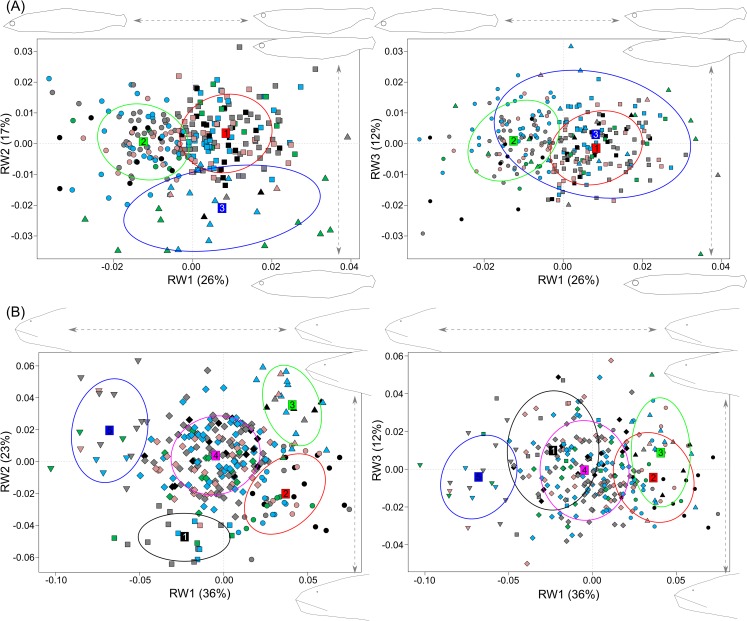
Association between morphological clusters and genetically-differentiated clusters of lake trout present in Mistassini Lake. Morphological clusters are identified by symbol shape and ellipses which represent 67% of that cluster’s variation and genetically-differentiated clusters are identified by symbol colour. (A) RW1 vs. 2 and 1 vs. 3 (55%) of body shape variation. Morphological shifts for RW1 (26%) correspond to the slope of the snout, lower jaw and dorsal and belly curvature; RW2 (17%) correspond to slope of top of cranium and dorsal side, length and depth of caudal peduncle; RW3 (12%) correspond to body depth, eye position and size. (B) RW 1 vs. 2 and 1 vs. 3 (71%) of head shape variation. Morphological shifts for RW1 (36%) correspond to length of head and snout, eye position and length of upper jaw; RW2 (23%) correspond to head depth and bluntness of snout; RW3 (12%) correspond to slope of lower and upper jaw. Inset images are visualizations of the shape at the most extreme end of each relative warp.

The seven body RWs explained 78% and the first three head RWs explained 70% of the total variation (34, 28 RWs, respectively). Major shape differences are visualized in Fig 3 and included the slope of the snout and lower jaw, dorsal and belly curvature (body RW1; 36%), slope of top of cranium and dorsal side, length and depth of caudal peduncle (body RW2; 17%), body depth, eye size (body RW3; 12%), length of head and snout, eye position, length of upper jaw (head RW1; 36%), head depth and bluntness of snout (head RW2; 23%) and slope of lower and upper jaw (head RW3; 12%).

MCLUST defined three separate body shape clusters (B1-3) with a ΔBIC = 42, and five head shape clusters (H1-5) with a ΔBIC = 6 (Table S4.1 in S4 File; Fig 3), and it recommended carrying forward the first seven body RWs body and three head RWs for further analyses. Consensus shapes of the three body clusters did not differ greatly, however, B1 (n = 144) had a long and steep lower jaw and long and narrow caudal peduncle, B2 (n = 111) had a shorter head with a steep cranium top and a long and narrow caudal peduncle, whereas B3 (n = 26) had the greatest body depth, smaller eyes and a thicker and shorter caudal peduncle (Fig S4.2A in S4 File). Morphological variation was greater among head shape clusters. H1 (n = 19) had the bluntest snout and shortest upper jaw. H2 (n = 45) had the longest head and a less blunt and more streamlined snout. H3 (n = 22) was the most streamlined with the longest upper jaw. H4 (n = 177) was very similar to H2 but with a slightly greater depth and longer upper jaw, and H5 (n = 18) had the shortest and deepest head with eyes located most dorsally (Fig S4.2B in S4 File). The DFA analysis resulted in 85% and 92% correct morphological assignment for body and head clusters, respectively (Table S4.2A,B in S4 File).

#### Body size and colouration

Mass varied among genetic clusters (*F*_4,515_ = 24.88, *P* < 0.001). Cluster 4 individuals were larger than all others (all *P* < 0.001); cluster 3 was larger than clusters 1, 2, and 5 (pairwise *t*-tests; *P* < 0.001–0.02; Fig S4.3A in S4 File). Both total length (TL) and the TL*population interaction had a significant effect on mass (*F*_9,437_ = 575.2, *P* < 0.001). The slope for the mass/length relationship of cluster 4 was significantly steeper than cluster 1, 2 and 3 (pairwise *t*-test; *P* < 0.003–0.006; Fig S4.3B in S4 File).

Genetic clusters varied in colouration type frequencies ($X_{16}^{2}$ = 130.91, *P* < 0.001). Clusters 2, 3, and 5 were predominately (between 73–80%) of black colouration (Fig 1B), whereas clusters 1 and 4 contained between 69 and 72% of lighter (dark silver, silver or light) coloured trout.

### Associations between genetic, morphological and ecological differentiation

#### Genetic & morphological or ecological associations

Genetic clusters were associated with body and head clusters (body: $X_{8}^{2}$ = 38.1, *P* < 0.001; head: $X_{16}^{2}$ = 38.1, *P* < 0.002) but these relationships were best described as weak (body: Cramer’s V = 0.26; head: Cramer’s V = 0.18). Each morphological cluster contained individuals in varying frequencies from all genetic clusters (Fig 3).

The selected RDA model included depth, basin and sector (R^2^_adj_ = 0.12). A global permutation test revealed relationships between ecological variables and genetic clustering (*P* = 0.001). A subsequent canonical axes permutation test revealed that only the first three RDA axes explained this relationship (all *P* = 0.001). RDA1 (50% of the variation) was driven primarily by depth and was especially important for distinguishing cluster 1 as occupying deeper water than all other clusters (Fig S8.1 in S8 File). RDA2 (37%) was primarily driven by individuals located in the eastern basin and sectors, and distinguished primarily cluster 5. RDA3 (12%) was primarily driven by individuals located in the western basin and sectors and was most important for distinguishing clusters 2 and 4. A supplementary, pairwise comparison of the least square mean depth showed that cluster 1 was captured at greater depths compared to all other clusters (Fig 1C). Fishing effort-corrected spatial frequencies also exemplified that clusters 1 and 5 were captured disproportionately more in the eastern than western basin (GLM, *P* < 0.001); clusters 3 and 4 showed the converse pattern (both *P* < 0.001), whereas cluster 2 was captured in equal numbers among basins (*P* = 0.886; Fig 1D).

As depth influenced genetic clustering, we also examined whether it affected morphological clustering. Depth was only a weak predictor of body shape cluster (linear model, *F*_2,202_ = 3.5, *P* = 0.033); only individuals in cluster B1 were captured at a greater mean depth compared to B2 individuals (pairwise *t*-tests, *P* = 0.027; Fig S8.2A in S8 File). Conversely, depth was a good predictor of head shape cluster (linear model, *F*_4,200_ = 7.4, *P* < 0.001). Cluster H5 individuals were captured at a greater mean depth than all other morphological clusters (pairwise *t*-tests, all *P* < 0.025; Fig S8.2B in S8 File); cluster H4 was captured more deeply than clusters H2 and H3 (*P* = 0.030 and 0.011 respectively) and cluster H1 was captured more deeply than cluster H3 (*P* = 0.029).

#### Genetic & fish community structure associations

Genetic clusters were associated with different quantities of bycatch of other fish species (*P* < 0.001), though the association was weak (Cramer’s V = 0.168). Cluster 1 was rarely captured with other fish species, clusters 2 and 3 were associated with burbot (*Lota lota*) and Lake whitefish, cluster 5 with walleye, and cluster 4 with all bycatch species (Fig S8.3 in S8 File).

#### Mantel tests: genetic differentiation vs. morphological or ecological differentiation

**Fig 4:**
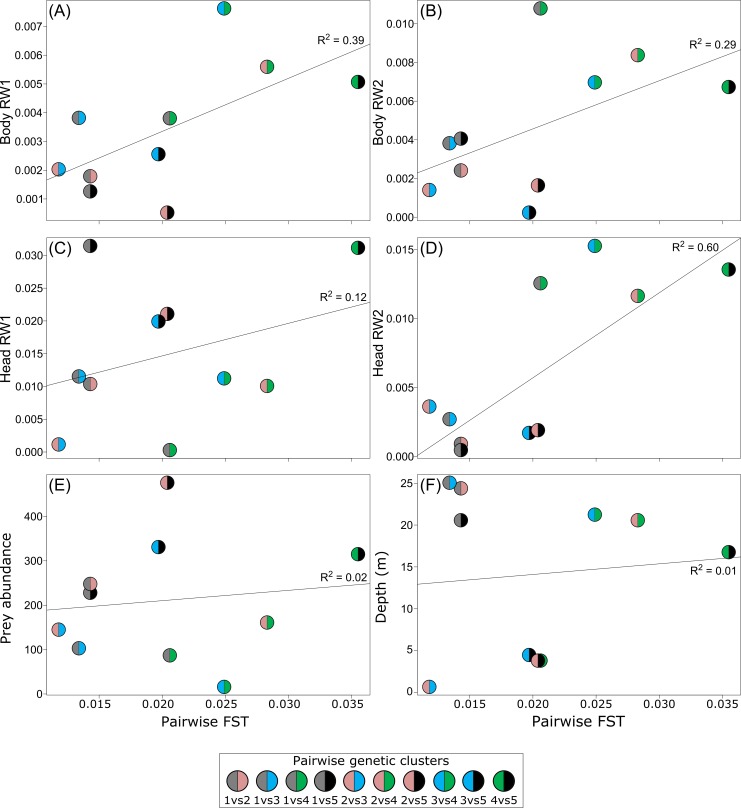
Visualization of Mantel tests: genetic distance vs. morphological and ecological variables. Shown are comparisons of genetic distance (pairwise F_ST_) vs. (i) the absolute difference in mean relative warp (RW) score for the first two RWs for body (A, B) and head morphology (C, D); (ii) absolute difference in prey abundance (E); and (iii) the absolute difference in median depth (m) (F). Body RW1 and 2 represent 26% and 17% of the total variation; whereas head RW1 and 2 represent 36% and 23% of the total variation. The remaining visualizations of Mantel tests can be seen in Fig S8.4 in S8 File.

There was little evidence for a relationship between cluster pairwise F_ST_ and the extent of morphological, water depth and prey differences (Table S8.1 in S8 File). Relationships only existed between F_ST_ and the differences in the observed colour frequencies of brown and black (*P* = 0.008 and 0.017 respectively). Specifically, pairwise F_ST_ was positively associated with both dark colours in which clusters with the greatest genetic differentiation also exhibited the greatest absolute difference in the frequencies of individuals that were black and brown. Additionally, some evident positive trends between increasing F_ST_ and increasing morphological differences were detected (body: RW1, RW2, RW5, RW6; head: RW1, RW2; Fig 4; Fig S8.4 in S8 File).

## Discussion

We detected striking phenotypic variation yet low genetic differentiation in lake trout occupying a large, pristine, postglacial lake. Genetic clustering was (i) influenced primarily by depth and less so by lake geography (basin, sector); (ii) associated with some variation in body size, colouration, and fish community structure, but was (iii) inconsistently associated with body/head shape differences linked to trophic ecology and locomotor mode. Rather, genetic clusters displayed high morphological variation, and all contained individuals belonging to the here-identified three body shape clusters or five head shape clusters.

### Associations between phenotypic variation, genetic differentiation and the environment; comparisons with other large, postglacial lakes

One obvious phenotype-environment association detected was camouflage body colouration and depth. In contrast to other, clear postglacial lakes inhabited by lake trout morphs, Mistassini is tea-coloured with a relatively shallow secchi depth (~ 9 m; [72]). Correspondingly, genetic clusters associated with shallower waters (#2, 3, 5; median = 5–10 m), mid-depths (#4; median = 27 m) and deeper areas (#1; median = 30 m) were, respectively, predominately black and brown in colouration, a mix of all colours, and frequently silver or white. Second, clusters with the largest (#4) and smallest (#1) body sizes were most frequently captured, respectively, at mid-depths associated with steep slopes harbouring all prey species and with less abundant prey species.

Functionality of different head shapes also corresponded somewhat with depth. Lake trout with dorsally located eyes, blunt snouts and short heads (H5) were routinely captured in deep water (median = 142 m), whereas individuals with more streamlined heads (H1 –H4) were captured in shallower water (median = 9–27 m); the former head shape is specialized for benthic foraging or more vertical oriented piscivory, the latter is associated with piscivory or insectivorous preferences in shallower waters [9]. Body shape differences between morphological clusters were less obvious and characterized by changes in body depth and caudal peduncle dimensions. Individuals captured in deeper water (B1 and B2; median = 33 and 45 m respectively) had long and narrow caudal peduncles which is needed for sustained, pelagic swimming [73]. Conversely, trout captured in shallower water (B3; median = 31 m) had the greatest body depth and much thicker and shorter caudal peduncles—traits usually linked to enhanced manoeuverability in more complex habitat [74], burst swimming and acceleration [73].

These general body shape-depth/habitat associations have similarities and differences with other lake trout morphs. The shallow-water, streamlined, long and narrow caudal peduncle ‘lean’ morph in other lakes [14] is similar to some of Mistassini’s B1 and B2 (and shallow-water genetic clusters 2, 3 and 5), and likely derived for pelagic swimming. Two deepwater morphs identified in other lakes–small-bodied ‘humpers’ with large, dorsally positioned eyes and typically found on steep slopes, and deeper-bodied ‘siscowets’ that have a sloping snout and thick and short caudal peduncles [14]–have shared similarities to morphological cluster H5 and large sized individuals of genetic cluster 4.

Despite some evidence for phenotypic-environment associations and drift-migration equilibrium conditions, we found only weak support for positive relationships between genetic and ecological or morphological differentiation and hence a putatively adaptive basis for occupying distinct habitat niches. Although some genetic clusters varied slightly in the frequencies of different morphologies, they all contained individuals exhibiting all three body types and all five head shape types. Furthermore, colouration and depth associations were not absolute: all five clusters contained individuals with each identified main colour and were captured in areas of differing fish-community structure. When compared to collated data from other large, postglacial lakes harbouring lake trout morphs, such relatively weak or little association between morphological and genetic differentiation appears common (Table 1).

**Table 1:** A comparison of lake physical attributes and sympatric differentiation of lake trout as currently described in the literature.

	ML (this study)	GBL	GSL	LS	AL
Surface area (km^2^)	2,335[23]	31,328[20]	28,568[22]	82,100[14]	792[21]
Depth; mean/max (m)	75 / 183[75]	90 / 450[20]	73 / 614[76]	147 / 406[14]	86 / 283[77]
Secchi depth (m)	9[72]	20–30[76,78]	~ 9[76]	15–20[79]	10[80]
# of basins	2	5[20]	2[22]	2	4[21]
# of fish species	12	15[20]	21[14]	87[14]	10[80]
Shoreline distance (km)	1,509 (+ 967 isl.)	2,719 (+ 824 isl.)[18,78]	3,057[14]	2,938[14]	391 (+ 214 isl.)
# of mtDNA lineages	4[26]	1[81]	3[26]	4[26]	2[21]
# of genetically diff. clusters	5	2[19]	Not tested to date	3[17]	3‡[21]
# of morph clusters	3 body, 5 head	3–4[20,81]	3[14]	4[14]	2[21]
Ass. btw morph and genetic?	Weak	None[20]–some[19]	Not tested to date	Some[17]	None[21]
Genetic diff. (global F_ST_)	0.017–0.028	0.008[19]	Not tested to date	0.024–0.033[17]	0.022[21]
$\bar{AR}$ (# of loci)	29.51 (19)	8.30 (22)[19]	Not tested to date	3.01 (9)[14]	11.8 (8)[21]
‘Shallow’ body morphology (0-30m)	1. Lean, streamlined, dark (black, brown)	1. Streamlined, small, dark/silvery 2. ‘Lean-like’, large, silvery 3. Stream-lined, dark, red fins 4. ‘Redfin-like’, darker, red fins[14]	1. 'Lean-like', streamlined, large, light*[22]	1. ‘Lean’: large-bodied, silvery or light[14,60]	1. ‘Lean-like’ morphs (streamlined, silvery)*[21]
‘Mid-depth’ body morphology (30-100m)	1. Large, deep-bodied, various colouration	No data to date	1. ‘Siscowet-like’, deep-body, large, dark^†^ 2. Deep body^†^[14,22]	1. ‘Humper’: small, narrow-bodied, silvery or light colour 2. ‘Redfin’: robust, large/deep-bodied, dark, red/yellow fins[14,60]	1. ‘Siscowet-like’, deeper body^§^[21]
‘Deepwater’ body morphology (>100m)	1. Small, deep-bodied, light or silvery	No data to date	No LT captured	1. ‘Siscowet’: large, deep-bodied, dark colour[14,60]	

Mistassini Lake (ML), Great Bear Lake (GBL), Great Slave Lake (GSL), Lake Superior (LS) and Atlin Lake (AL).

* Captured between 0–50 m

†Captured between > 50–100 m

‡ Three genetically-differentiated populations and their descriptive statistics were described in Northrup et al. [21] for the Atlin-Tagish Lake system (two lakes interconnected by river in which lake trout are also found)

§captured between 70–150 m.

### Factors influencing low genetic differentiation, comparisons with other postglacial fishes

Where striking examples of multiple, sympatrically-occurring morphs of lake trout have been described, they are almost universally found in very large postglacial lakes [82]. This specificity for large lakes probably relates to (i) the large *N*_e_ that a large lake size confers towards sustaining multiple evolving populations of a large-bodied predator fish, and (ii) the different habitat niches available in larger lakes that lake trout can exploit, including shallow and deep water habitat, extensive shoreline distance, multiple basins and multiple prey fish species (Table 1). Notably, however, genetic differentiation among documented morphs is always low or relatively weak (range of global F_ST_ of 0.008–0.022; [17,19,21]; this study), and relative to previously studied fishes in Mistassini Lake (brook trout, global F_ST_ = 0.071; walleye, global F_ST_ = 0.045; [25,83]). At least four specific aspects of the ecology, behaviour and genetics of lake trout might facilitate such low differentiation.

#### Reduced natal fidelity and lifecycle complexity

Compared to the tributary spawning nature of brook trout and walleye, which generates considerable spatial isolation (tens to hundreds of kilometres) and a more complex lifecycle, Mistassini lake trout spawn along lake shorelines, and local fishing experts have described the spawning sites of various morphs in close geographic proximity (K. Marin 2013 pers. comm.). Although natal fidelity is well documented among postglacial lake-dwelling salmonids [84], the evidence for natal fidelity in lake trout is weak and even contradictory [85,86]; the close proximity of spawning site locations in Mistassini Lake may therefore facilitate gene flow and disfavour reproductive isolation in the long-term.

#### Constraints from gene flow

Consideration of *N*_e_ and *m* revealed significant asymmetries in *m* among genetic clusters, and hence the possibility that gene flow constrains adaptive divergence [87] in certain habitat niches for lake trout. Namely, *m* was significantly higher from the large genetic cluster occupying deeper waters (#1) to large and small clusters occupying mid-depths and shallow waters (#2, 4, 5). Adaptive divergence for occupying some habitat niches in shallower waters may thus be constrained; alternatively, selection against migrants may be stronger against individuals from shallower than deeper genetic clusters.

#### Constraints from phenotypic plasticity

Whereas defined genetic clusters exhibited summer depth preferences, individuals from different clusters were captured throughout the water column. The striking morphological variation was more continuous within than among genetic clusters, suggesting a highly plastic propensity for occupying multiple habitat niches. Habitat use among individual lake trout has indeed been found to be highly variable but consistent between years [88–90].

Phenotypic plasticity is thought to both facilitate and constrain adaptive divergence and reproductive isolation during the process of ecological speciation [2,91]. Sympatric differentiation in lake trout might be quite unique in exemplifying how plasticity may constrain further steps towards ecological speciation. For example, in Mistassini Lake, low genetic differentiation was unrelated to a potential downward bias in F_ST_ and a lack of migration-drift equilibrium, but rather, related to a high contemporary gene flow and large *N*_e_. And, in contrast to other cases in north temperate fishes [92,93], including co-generic Arctic charr [8], remarkable phenotypic variation in Mistassini lake trout and in other lakes does not result in appreciable genetic segregation nor a clear correspondence between different phenotypes and different habitat niches, despite evidence for the availability of the latter. Such discontinuous adaptive variation with minor reproductive isolation implies that i) selection against migrants or hybrids between diversifying lake trout populations is not very strong, ii) morphological plasticity is not very costly in this species, and/or iii) the extent of adaptive divergence might be constrained by adaptive plasticity [2].

If low genetic differentiation in sympatric lake trout is constrained by plasticity, then a key question is why plasticity might more readily constrain later steps of ecological speciation in this species relative to congeneric Arctic charr. One possibility is that the evolution of lake trout was initially born out of specialization for terminal piscivory in cold-water lakes [82], whereas Arctic charr do not exhibit such near ubiquity in trophic specialization [6]. Such specialization might act as a developmental limitation to further diversification through phylogenetic niche conservatism, and may in part explain the rarity of genetically-distinguishable morphs in lake trout. A second possibility is that species differences in life history might have evolved in response to previous glacial cycles. The evolution of lake trout, unlike that of Arctic charr, was refined to the freshwater environment as opposed to marine and freshwaters [82]. The evolution of the anadromous life history stage of Arctic charr may have therefore played a critical role in the high precision of natal homing in this species [94]. Hence, adaptive plasticity and adaptive divergence might be more coupled, increasing the likelihood of greater genetic segregation arising where different habitat niches exist. Collectively, the unique evolutionary history of two congeneric species may allow plasticity to act as a constraint to ecological speciation at early stages in one species (lake trout) but help facilitate it at later stages in another (Arctic charr).

### Genetic diversity and evolutionary origins of sympatric lake trout morphs

This study on the complete spatial distribution of Mistassini lake trout (n = 636) and an independent study that screened Mistassini lake trout of unknown location (n = 46) using the same 19 microsatellite loci [95], have found correspondingly high genetic diversity (mean N_A_ = 29.51 and 17.26, respectively). Indeed, we found no evidence that Mistassini lake trout have experienced bottlenecks (S9 File) and the pristine nature of the lake probably means that the large *N*_e_ of inferred genetic clusters has not been reduced since colonization. Mistassini morphs therefore display high genetic diversity relative to morphs in other large, postglacial lakes (mean A_R_ = 3.01–11.80; Table 1). However, studies on other lakes used fewer (exception: Great Bear) and different microsatellite loci. Furthermore, lower genetic diversity in other lakes may be due to incomplete spatial sampling, lower numbers of colonizing mtDNA lineages (one in Great Bear vs. up to four in Mistassini [19,26]; or historical reductions from overfishing, invasive species and/or intensive stocking (i.e., Lake Superior) [96].

We found that genetic differentiation among clusters accounting for the mutational properties of microsatellites (R_ST_) exceeded global F_ST_ (S3 File), and hence affirmed previous work [26] that multiple lake trout lineages colonized Mistassini Lake. Indeed, Mistassini’s age is too young (8,000 years) and the number of generations passed are too few (667; 12 year generation time; [28,29]) to have generated this mutationally-driven differentiation entirely within sympatry. Yet an equilibrium between drift and gene flow has likely been reached since lake colonization, due to relatively high contemporary gene flow compared to *N*_e_ (Fig S7.1; Table S7.2 in S7 File). Because at least one lake (Great Bear) was postglacially colonized by a single mtDNA lineage [19,26], an allopatric phase does not appear to be a prerequisite for sympatric differentiation in lake trout as observed in some postglacial fishes (e.g., [97,98]). However, as our study reveals, individuals from different lake trout lineages have a propensity to occupy and exploit multiple available habitat niches.

### Conclusion: early-stage ecological speciation and conservation implications

**Fig 5:**
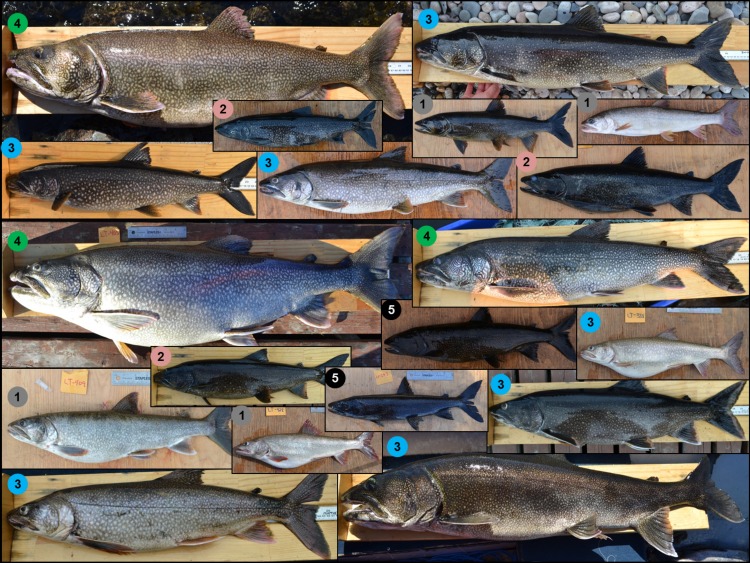
Morphological variation among individual lake trout and across all genetically-demarcated clusters within Mistassini Lake, Québec. Coloured circles and numbers represent the genetically-demarcated clusters that individual lake trout were assigned to.

In several large, postglacial lakes, lake trout exhibit non-random structuring or complexes of weakly diversifying populations with often striking phenotypic variation associated or partially associated with depth, spatial distribution and/or resource exploitation (Fig 5; Table 1); these morphs do not easily correspond to the classical benthic-limnetic morphs found in other postglacial fishes (e.g., [2,5,97]). In a remarkably pristine system (Mistassini Lake), our study revealed likely factors influencing the progress or lack of progress at earlier stages of population differentiation in the context of ecological speciation: genetic clusters appear to harbour considerable phenotypic plasticity, their habitat use distinctions were not clearly defined with individuals patchily occupying different habitat niches, reproductive habitats of different morphs were in close spatial proximity, and any adaptive divergence generated from differential habitat use appears to only generate weak reproductive isolation. Plasticity in particular, may act as a constraint to further diversification and place lake trout at the early-stages of the ecological speciation continuum [2].

Whether lake trout morphs have ‘plateaued’ in their steps towards true ecological speciation, or whether further adaptive divergence and strengthening of reproductive isolation is still to come in a future context, the maintenance of such differentiation may be important for species persistence in the face of changing environments [5]. Indeed, lake trout are found in tens of thousands of lakes but sympatric morphs have been documented in only a handful. Our research suggests that different morphs may be more or less susceptible to different harvesting techniques (gillnetting vs. angling). Their occupancy of large, deep lakes means that such lakes act as reservoirs of genetic diversity and cold water refuge habitat for the species in the face of future climate change. As a terminal predator, conservation plans for lake trout or for large postglacial ecosystems may benefit from a joint consideration of the role and survival of both prey, competitors (e.g., brook trout, walleye, whitefish, etc.) and conspecifics that thrive across a diversity of habitats, to maintain the processes generating biologically significant differentiation.

## Supporting Information

S1 File Loci and polymerase chain reaction (PCR) information. (PDF)

S2 File Supplemental genetic clustering approaches. (PDF)

S3 File Genetic differentiation by stepwise mutation (R_ST_). (PDF)

S4 File Supplemental morphological figures and tables. (PDF)

S5 File Multinomial regression analysis. (PDF)

S6 File Supplemental genetic data of lake trout in Mistassini Lake. (PDF)

S7 File Effective population size (*N*_*e*_) and gene flow Mistassini lake trout.
(PDF)

S8 File Supplemental tables and figures for associations between genetic, morphological and ecological differentiation. (PDF)

S9 File Assessment of recent bottlenecks of Mistassini lake trout. (PDF)

## References

[1] Rundle HD, Nosil P. Ecological speciation. Ecol Lett. 2005;8: 336–352. 10.1111/j.1461-0248.2004.00715.x

[2] Hendry AP. Ecological speciation! Or the lack thereof? Can J Fish Aquat Sci. 2009;66: 1383–1398. 10.1139/F09-074

[3] Taylor EB, Tamkee P, Keeley ER, Parkinson EA. Conservation prioritization in widespread species: the use of genetic and morphological data to assess population distinctiveness in rainbow trout (*Oncorhynchus mykiss*) from British Columbia, Canada. Evol Appl. 2011;4: 100–115. 10.1111/j.1752-4571.2010.00136.x 25567956 PMC3352517

[4] Fraser DJ. The emerging synthesis of evolution with ecology in fisheries science. Can J Fish Aquat Sci. 2013;70: 1417–1428.

[5] Taylor EB. Species pairs of north temperate freshwater fishes: Evolution, taxonomy, and conservation. Rev Fish Biol Fish. 1999;9: 299–324.

[6] Klemetsen A. The charr problem revisited: exceptional phenotypic plasticity promotes ecological speciation in postglacial lakes. Freshw Rev. 2010;3: 49–74. 10.1608/FRJ-3.1.3

[7] Fraser DJ, Calvert AM, Bernatchez L, Coon A. Multidisciplinary population monitoring when demographic data are sparse: a case study of remote trout populations. Ecol Evol. 2013;3: 4954–4969. 10.1002/ece3.871 24455128 PMC3892360

[8] Gíslason D, Ferguson MM, Skúlason S, Snorrason SS. Rapid and coupled phenotypic and genetic divergence in Icelandic Arctic char (*Salvelinus alpinus*). Can J Fish Aquat Sci. 1999;56: 2229–2234. 10.1139/f99-245

[9] Jonsson B, Jonsson N. Polymorphism and speciation in Arctic charr. J Fish Biol. 2001;58: 605–638. 10.1006/jfbi.2000.1515

[10] Berner D, Grandchamp AC, Hendry AP. Variable progress toward ecological speciation in parapatry: Stickleback across eight lake-stream transitions. Evolution (N Y). 2009;63: 1740–1753. 10.1111/j.1558-5646.2009.00665.x 19228184

[11] Rogers S, Bernatchez L. The Genetic Architecture of Ecological Speciation and the Association with Signatures of Selection in Natural Lake Whitefish (*Coregonus* sp. Salmonidae) Species Pairs. Mol Biol Evol. 2007;24: 1423–1438. 10.1093/molbev/msm066 17404398

[12] Hendry AP. Adaptive divergence and the evolution of reproductive isolation in the wild: An empirical demonstration using introduced sockeye salmon. Genetica. 2001;112–113: 515–534. 10.1023/A:1013367100865 11838786

[13] Blackie CT, Weese DJ, Noakes DLG. Evidence for resource polymorphism in the lake charr (*Salvelinus namaycush*) population of Great Bear Lake, Northwest Territories, Canada. Écoscience. 2003;10: 509–514.

[14] Muir AM, Hansen MJ, Bronte CR, Krueger CC. If Arctic charr Salvelinus alpinus is “the most diverse vertebrate”, what is the lake charr Salvelinus namaycush? Fish Fish. 2015; 1–14. 10.1111/faf.12114

[15] Krueger CC, Ihssen PE. Review of Genetics of Lake Trout in the Great Lakes: History, Molecular Genetics, Physiology, Strain Comparisons, and Restoration Management. J Great Lakes Res. Elsevier; 1995;21: 348–363. 10.1016/S0380-1330(95)71109-1

[16] Moore SA, Bronte CR. Delineation of Sympatric Morphotypes of Lake Trout in Lake Superior. Trans Am Fish Soc. 2001;130: 1233–1240.

[17] Page KS, Scribner KT, Burnham-Curtis M. Genetic Diversity of Wild and Hatchery Lake Trout Populations: Relevance for Management and Restoration in the Great Lakes. Trans Am Fish Soc. 2004;133: 674–691. 10.1577/T03-007.1

[18] Alfonso NR. Evidence for two morphotypes of lake charr, *Salvelinus namaycush*, from Great Bear Lake, Northwest Territories, Canada. Environ Biol Fishes. 2004;71: 21–32. 10.1023/B:EBFI.0000043176.61258.3d

[19] Harris LN, Chavarie L, Bajno R, Howland KL, Wiley SH, Tonn WM, et al Evolution and origin of sympatric shallow-water morphotypes of Lake Trout, *Salvelinus namaycush*, in Canada’s Great Bear Lake. Heredity (Edinb). 2014; 1–13. 10.1038/hdy.2014.74 PMC4815598 25204304

[20] Chavarie L, Howland K, Harris L, Tonn W. Polymorphism in lake trout in Great Bear Lake: intra-lake morphological diversification at two spatial scales. Biol J Linn Soc. 2014;114: 109–125. 10.1111/bij.12398

[21] Northrup S, Connor M, Taylor EB. Population structure of lake trout (Salvelinus namaycush) in a large glacial-fed lake inferred from microsatellite DNA and morphological analysis. Can J Fish Aquat Sci. 2010;67: 1171–1186. 10.1139/F10-054

[22] Zimmerman MS, Krueger CC, Eshenroder RL. Phenotypic Diversity of Lake Trout in Great Slave Lake: Differences in Morphology, Buoyancy, and Habitat Depth. Trans Am Fish Soc. 2006;135: 1056–1067. 10.1577/T05-237.1

[23] Statistics Canada. Principal lakes, elevation and area, by province and territory [Internet]. 2005. Available: http://www.statcan.gc.ca/tables-tableaux/sum-som/l01/cst01/phys05-eng.htm

[24] Fraser DJ, Bernatchez L. Adaptive migratory divergence among sympatric brook charr populations. Evolution (N Y). 2005;59: 611–624. 10.1554/04-346 15856703

[25] Dupont P-P, Bourret V, Bernatchez L. Interplay between ecological, behavioural and historical factors in shaping the genetic structure of sympatric walleye populations (*Sander vitreus*). Mol Ecol. 2007;16: 937–951. 10.1111/j.1365-294X.2006.03205.x 17305852

[26] Wilson CC, Hebert PD. Phylogeography and postglacial dispersal of lake trout (*Salvelinus namaycush*) in North America. Can J Fish Aquat Sci. 1998;55: 1010–1024. 10.1139/f97-286

[27] Zimmerman MS, Krueger CC, Eshenroder RL. Morphological and Ecological Differences Between Shallow- and Deep-water Lake Trout in Lake Mistassini, Quebec. J Great Lakes Res. 2007;33: 156–169. 10.3394/0380-1330(2007)33[156:MAEDBS]2.0.CO;2

[28] Hansen MJ, Nate NA, Krueger CC, Zimmerman MS, Kruckman HG, Taylor WW. Age, Growth, Survival, and Maturity of Lake Trout Morphotypes in Lake Mistassini, Quebec. Trans Am Fish Soc. 2012;141: 1492–1503. 10.1080/00028487.2012.711263

[29] Dubois A, Lagueux R. Comparative study of the age as determined by scales and otoliths of the lake trout (Salvelinus namaycush) of Mistassini Lake, Quebec. Nat Can. 1968; 907–928.

[30] van Oosterhout C, Hutchinson WF, Wills DP, Shipley P. MICRO-CHECKER: software for identifying and correcting genotyping errors in microsatellite data. Mol Ecol Notes. 2004;4: 535–538. 10.1111/j.1471-8286.2004.00684.x

[31] Goudet J. FSTAT: a program to estimate and test gene diversities and fixation indices [Internet]. 2002. Available: http://www.unil.ch/izea/softwares/fstat.html

[32] Excoffier L, Lischer HEL. Arlequin suite ver 3.5: A new series of programs to perform population genetics analyses under Linux and Windows. Mol Ecol Resour. 2010;10: 564–567. 10.1111/j.1755-0998.2010.02847.x 21565059

[33] Rousset F. GENEPOP’007: a complete re-implementation of the GENEPOP software for Windows and Linux. Mol Ecol Resour. 2008;8: 103–106. 10.1111/j.1471-8286.2007.01931.x 21585727

[34] Pritchard JK, Stephens M, Donnelly P. Inference of population structure using multilocus genotype data. Genetics. 2000;155: 945–959. 10835412 10.1093/genetics/155.2.945 PMC1461096

[35] Evanno G, Regnaut S, Goudet J. Detecting the number of clusters of individuals using the software STRUCTURE: a simulation study. Mol Ecol. 2005;14: 2611–20. 10.1111/j.1365-294X.2005.02553.x 15969739

[36] Earl DA, vonHoldt BM. STRUCTURE HARVESTER: a website and program for visualizing STRUCTURE output and implementing the Evanno method. Conserv Genet Resour. 2012;4: 359–361. 10.1007/s12686-011-9548-7

[37] Jakobsson M, Rosenberg NA. CLUMPP: a cluster matching and permutation program for dealing with label switching and multimodality in analysis of population structure. Bioinformatics. 2007;23: 1801–1806. 10.1093/bioinformatics/btm233 17485429

[38] Anderson EC, Dunham KK. The influence of family groups on inferences made with the program Structure. Mol Ecol Resour. 2008;8: 1219–1229. 10.1111/j.1755-0998.2008.02355.x 21586009

[39] Wang J. Sibship Reconstruction from Genetic Data with Typing Errors. Genetics. 2004;166: 1963–1979. 10.1534/genetics.166.4.1963 15126412 PMC1470831

[40] Weir BS, Cockerham CC. Estimating F-Statistics for the Analysis of Population Structure. Evolution (N Y). 1984;38: 1358–1370. 10.1111/j.1558-5646.1984.tb05657.x 28563791

[41] Fraser DJ, Bernatchez L. Allopatric origins of sympatric brook charr populations: colonization history and admixture. Mol Ecol. 2005;14: 1497–1509. 10.1111/j.1365-294X.2005.02523.x 15813787

[42] Hardy OJ, Charbonnel N, Fréville H, Heuertz M. Microsatellite allele sizes: a simple test to assess their significance on genetic differentiation. Genetics. 2003;163: 1467–82. 12702690 10.1093/genetics/163.4.1467 PMC1462522

[43] Balloux F, Goudet J. Statistical properties of population differentiation estimators under stepwise mutation in a finite island model. Mol Ecol. 2002;11: 771–783. 10.1046/j.1365-294X.2002.01474.x 11972763

[44] Gaggiotti OE, Lange O, Rassmann K, Gliddon C. A comparison of two indirect methods for estimating average levels of gene flow using microsatellite data. Mol Ecol. 1999;8: 1513–1520. 10.1046/j.1365-294X.1999.00730.x 10564457

[45] Jombart T, Devillard S, Dufour A-B, Pontier D. Revealing cryptic spatial patterns in genetic variability by a new multivariate method. Heredity (Edinb). 2008;101: 92–103. 10.1038/hdy.2008.34 18446182

[46] Jombart T. adegenet: a R package for the multivariate analysis of genetic markers. Bioinformatics. 2008;24: 1403–1405. 10.1093/bioinformatics/btn129 18397895

[47] R Core Team. R: A language and environment for statistical computing Vienna, Austria: R Foundation for Statistical Computing; 2013.

[48] Moran P. The Interpretation of Statistical Maps. J R Stat Soc. 1948;10: 243–251.

[49] Upton G, Fingleton B. Spatial Data Analysis by Example: Point Pattern and Quantitative Data. Volume 1. Wiley Series in Probability and Mathematical Statistics; 1985.

[50] Guillot G, Estoup A, Mortier F, Cosson JF. A Spatial Statistical Model for Landscape Genetics. Genetics. 2005;170: 1261–1280. 10.1534/genetics.104.033803 15520263 PMC1451194

[51] Waples RS, Do C. LDNE: A program for estimating effective population size from data on linkage disequilibrium. Mol Ecol Resour. 2008;8: 753–756. 10.1111/j.1755-0998.2007.02061.x 21585883

[52] Waples RS, Antao T, Luikart G. Effects of Overlapping Generations on Linkage Disequilibrium Estimates of Effective Population Size. Genetics. 2014;197: 769–780. 10.1534/genetics.114.164822 24717176 PMC4063931

[53] Wilson GA, Rannala B. Bayesian Inference of Recent Migration Rates Using Multilocus Genotypes. Genetics. 2003;163: 1177–1191. 12663554 10.1093/genetics/163.3.1177 PMC1462502

[54] Rambaut A, Suchard M, Xie D, Drummond A. Tracer v1.6 [Internet]. 2014. Available: http://beast.bio.ed.ac.uk/Tracer

[55] Whitlock MC. Temporal Fluctuations in Demographic Parameters and the Genetic Variance among Populations. Evolution (N Y). 1992;46: 608–615. 10.2307/2409631 28568658

[56] Rohlf F. tpsDIG2 [Internet]. Department of Ecology and Evolution, State University of New York at Stony Brook; 2013. Available: http://life.bio.sunysb.edu/morph/soft-dataacq.html

[57] Zimmerman MS, Schmidt SN, Krueger CC, Vander Zanden MJ, Eshenroder RL. Ontogenetic niche shifts and resource partitioning of lake trout morphotypes. Can J Fish Aquat Sci. 2009;66: 1007–1018. 10.1139/F09-060

[58] Sheets DH. Integrated Morphometrics Package (IMP8): MakeFan [Internet]. 2014. Available: http://www.canisius.edu/~sheets/morphsoft.html

[59] Zelditch ML, Swiderski DL, Sheets DH, Fink WL. Geometric Morphometrics for Biologists: a Primer Elsevier; 2004.

[60] Muir AM, Bronte CR, Zimmerman MS, Quinlan HR, Glase JD. Ecomorphological Diversity of Lake Trout at Isle Royale, Lake Superior. Trans Am Fish Soc. 2014;143: 972–987. 10.1080/00028487.2014.900823

[61] Rohlf F. tpsRelw32 [Internet]. Department of Ecology and Evolution, State University of New York at Stony Brook; 2003. Available: http://life.bio.sunysb.edu/morph/soft-tps.html

[62] Bookstein F. Morphometric tools for landmark data: geometry and biology New York: Cambridge University Press; 1991.

[63] Fraser DJ, Weir LK, Darwish TL, Eddington JD, Hutchings JA. Divergent compensatory growth responses within species: Linked to contrasting migrations in salmon? Oecologia. 2007;153: 543–553. 10.1007/s00442-007-0763-6 17541646

[64] Fraley C, Raftery AE. MCLUST Version 3 for R: Normal Mixture Modeling and Model-Based Clustering. 2006.

[65] Fraley C, Raftery AE. Enhanced Model-Based Clustering, Density Estimation, and Discriminant Analysis Software: MCLUST. J Classif. 2003;20: 263–286. 10.1007/s00357-003-0015-3

[66] Hammer Ø, Harper DAT, Ryan PD. PAST: Paleontological statistics software package for education and data analysis. Palaeontol Electron. 2001;4: 9–18.

[67] Lenth R. lsmeans: Least-Squares Means. R package [Internet]. 2015. Available: http://cran.r-project.org/package=lsmeans

[68] Meyer D, Zeileis A, Hornik K. vcd: Visualizing Categorical Data. R package. 2015.

[69] Oksanen J, Blanchet FG, Kindt R, Legendre P, Minchin PR, O’Hara R, et al. vegan: Community Ecology Package. R package [Internet]. 2015. Available: http://cran.r-project.org/package=vegan

[70] Borcard D, Gillet F, Legendre P. Numerical Ecology with R. 2011.

[71] Benjamini Y, Hochberg Y. Controlling the false discovery rate: a practical and powerful approach to multiple testing. R Stat Soc Ser B. 1995;57: 289–300.

[72] Legendre P, Beauvais A. Niches et associations de poissons de lacs de la radissonie Quebecoise. Le Nat Can. 1978;105: 137–158.

[73] Webb P. Body Form, Locomotion and Foraging in Aquatic Vertebrates. Am Zool. 1984;24: 107–120. 10.1093/icb/24.1.107

[74] Kristjánsson BK, Skúlason S, Noakes DLG. Morphological segregation of Icelandic threespine stickleback (*Gasterosteus aculeatus* L). Biol J Linn Soc. 2002;76: 247–257. 10.1111/j.1095-8312.2002.tb02086.x

[75] International Lake Environment Committee Foundation (ILECF). World Lake Database: Lake Mistassini [Internet]. 1999 [cited 11 May 2013]. Available: http://wldb.ilec.or.jp/Lake.asp?LakeID=SNAM-095&RoutePrm=0:;4:load;

[76] Evans M. The large lake ecosystems of northern Canada. Aquat Ecosyst Heal Manag. 2000;3: 65–79. 10.1016/S1463-4988(99)00071-8

[77] Downie AJ. Drinking Water Source Quality Monitoring 2002–03. Atlin & Area: Atlin Lake, Warm Bay Road Spring, Fourth of July Creek and Groundwater. 2005.

[78] Johnson L. Physical and chemical characteristics of Great Bear Lake, Northwest Territories. J Fish Res Board Canada. 1975;32: 1871–1987.

[79] Water on the Web. Lake Ecology Primer: Light [Internet]. 2011 [cited 3 Dec 2014]. Available: http://www.waterontheweb.org/under/lakeecology/04_light.html

[80] Lindsey CC, Patalas K, Bodaly RA, Archibald CP. Glaciation and the physical, chemical and biological limnology of Yukon Lakes. Winnipeg, Manitoba; 1981.

[81] Chavarie L, Howland KL, Tonn WM. Sympatric Polymorphism in Lake Trout: The Coexistence of Multiple Shallow-Water Morphotypes in Great Bear Lake. Trans Am Fish Soc. 2013;142: 814–823. 10.1080/00028487.2013.763855

[82] Wilson CC, Mandrak NE. History and evolution of lake trout in Shield lakes: past and future challenges In: Gunn J, Steedman RJ, Ryder R, editors. Boreal Shield Watersheds: Lake Trout Ecosystems in a Changing Environment. CRC Press; 2003.

[83] Fraser DJ, Lippe C, Bernatchez L. Consequences of unequal population size, asymmetric gene flow and sex-biased dispersal on population structure in brook charr (*Salvelinus fontinalis*). Mol Ecol. 2004;13: 67–80. 10.1046/j.1365-294X.2003.02038.x 14653789

[84] Stewart IJ, Quinn TP, Bentzen P. Evidence for fine-scale natal homing among island beach spawning sockeye salmon, *Oncorhynchus nerka*. Environ Biol Fishes. 2003;67: 77–85. 10.1023/A:1024436632183

[85] Scott W, Crossman E. Freshwater Fishes of Canada. Bulletin 1. Fisheries Research Board of Canada; 1973.

[86] MacLean J, Evans D, Martin N. Survival, Growth, Spawning Distribution, and Movements of Introduced and Native Lake Trout (*Salvelinus namaycush*) in Two Inland Ontario Lakes. Can J Fish Aquat Sci. 1981;38: 1685–1700. 10.1139/f81-217

[87] Hendry AP, Taylor EB. How much of the variation in adaptive divergence can be explained by gene flow? An evaluation using lake-stream stickleback pairs. Evolution. 2004;58: 2319–2331. 10.1111/j.0014-3820.2004.tb01606.x 15562693

[88] Snucins EJ, Gunn JM. Coping with a Warm Environment: Behavioral Thermoregulation by Lake Trout. Trans Am Fish Soc. 1995;124: 118–123. 10.1577/1548-8659(1995)124<0118:CWAWEB>2.3.CO;2

[89] Sellers TJ, Parker BR, Schindler DW, Tonn WM. Pelagic distribution of lake trout (*Salvelinus namaycush*) in small Canadian Shield lakes with respect to temperature, dissolved oxygen, and light. Can J Fish Aquat Sci. 1998;55: 170–179. 10.1139/f97-232

[90] Morbey YE, Addison P, Shuter BJ, Vascotto K. Within-population heterogeneity of habitat use by lake trout *Salvelinus namaycush*. J Fish Biol. 2006;69: 1675–1696. 10.1111/j.1095-8649.2006.01236.x

[91] Lande R. Adaptation to an extraordinary environment by evolution of phenotypic plasticity and genetic assimilation. J Evol Biol. 2009;22: 1435–1446. 10.1111/j.1420-9101.2009.01754.x 19467134

[92] Taylor EB, Bentzen P. Evidence for Multiple Origins and Sympatric Divergence of Trophic Ecotypes of Smelt (*Osmerus*) in Northeastern North America. Evolution (N Y). 1993;47: 813–832. 10.2307/2410186 28567890

[93] Hendry AP, Bolnick DI, Berner D, Peichel CL. Along the speciation continuum in sticklebacks. J Fish Biol. 2009;75: 2000–2036. 10.1111/j.1095-8649.2009.02419.x 20738669

[94] Nordeng H. Char ecology. Natal homing in sympatric populations of anadromous Arctic char *Salvelinus alpinus* (L.): roles of pheromone recognition. Ecol Freshw Fish. 2009;18: 41–51. 10.1111/j.1600-0633.2008.00320.x

[95] Valiquette E, Perrier C, Thibault I, Bernatchez L. Loss of genetic integrity in wild lake trout populations following stocking: insights from an exhaustive study of 72 lakes from Québec, Canada. Evol Appl. 2014; 1–20. 25067947 10.1111/eva.12160 PMC4105915

[96] Baillie SM, Muir A, Scribner K, Bentzen P, Krueger C. Loss of genetic diversity and reduction of genetic distance among lake trout *Salvelinus namaycush* ecomorphs, Lake Superior 1959 to 2013. J Great Lakes Res. The Authors; 2016;42: 204–216. 10.1016/j.jglr.2016.02.001

[97] Bernatchez L, Dodson JJ. Allopatric Origin of Sympatric Populations of Lake Whitefish (*Coregonus clupeaformis*) as Revealed by Mitochondrial-DNA Restriction Analysis. Evolution (N Y). 1990;44: 1263–1271. 10.2307/2409287 28563883

[98] Taylor EB, McPhail JD. Historical contingency and ecological determinism interact to prime speciation in sticklebacks, *Gasterosteus*. Proc R Soc B Biol Sci. 2000;267: 2375–2384. 10.1098/rspb.2000.1294 PMC1690834 11133026

