# Supplementary material for: Striking Phenotypic Variation yet Low Genetic Differentiation in Sympatric Lake Trout (Salvelinus namaycush)
Source: PLoS One. 2016 Sep 28;11(9):e0162325. doi: 10.1371/journal.pone.0162325 (PMC5040267; doi:10.1371/journal.pone.0162325)
Supplement: S1 File — (PDF) [file pone.0162325.s001.pdf]

Loci and polymerase chain reaction (PCR) information

The DNA from the 636 individual lake trout was extracted and amplified at 19 highly polymorphic microsatellites [1–5]. These included the following loci arranged in 8 reactions including four uniplexes (*SfoD75*, *Sfo308*, *Sna07*, *Sna09*), one triplex (*Sfo226*, *Sna02*, *Sna03*), and three quadplexes (*Sna01*, *Sco200*, *Smm22*, *SSsp2201*; *Sna11*, *Sna12, Sna13*, *Sco215;* and *Sna06*, *Sna08*, *Sco202*, *Sna10*). Polymerase chain reaction (PCR) amplifications were carried out using a modified version of Valiquette et al. [6] and carried out in a 10 µl final volume: each reaction contained 5 to 25 ng DNA template, 1 µl TAQ buffer (1x), 0.2 mM dNTP, 2.0 mM MgSO4, 0.8 mg BSA, and forward (fluorescently labelled) and reverse primers at varying concentrations. Amplifications were performed using a T100 Thermal Cycler (BioRad) with an initial 4 min denaturing step at 95ºC, followed by 35 cycles of denaturation at 95ºC for 20 s, annealing at 58ºC for 20 s and extension at 72ºC for 15 s followed by a final extension at 72ºC for 5 min.

References

1. Perry GML, King TL, St-Cyr J, Valcourt M, Bernatchez L. Isolation and cross-familial amplification of 41 microsatellites for the brook charr (Salvelinus fontinalis). Mol Ecol Notes. 2005;5: 346–351. doi:10.1111/j.1471-8286.2005.00922.x

2. Rollins MF, Vu N V, Spies IB, Kalinowski ST. Twelve microsatellite loci for lake trout (Salvelinus namaycush). Mol Ecol Resour. 2009;9: 871–3. doi:10.1111/j.1755-0998.2008.02403.x

3. Dehaan PW, Ardren WR. Characterization of 20 highly variable tetranucleotide microsatellite loci for bull trout (Salvelinus confluentus) and cross-amplification in other Salvelinus species. Mol Ecol Notes. 2005;5: 582–585. doi:10.1111/j.1471-8286.2005.00997.x

4. Crane PA, Lewis CJ, Kretschmer EJ, Miller SJ, Spearman WJ, DeCicco AL, et al. Characterization and inheritance of seven microsatellite loci from Dolly Varden, Salvelinus malma, and cross-species amplification in Arctic char, S. alpinus. Conserv Genet. 2004;5: 737–741. doi:10.1007/s10592-004-1853-1

5. Paterson S, Piertney SB, Knox D, Gilbey J, Verspoor E. Characterization and PCR multiplexing of novel highly variable tetranucleotide Atlantic salmon (Salmo salar L.) microsatellites. Mol Ecol Notes. 2004;4: 160–162. doi:10.1111/j.1471-8286.2004.00598.x

6. Valiquette E, Perrier C, Thibault I, Bernatchez L. Loss of genetic integrity in wild lake trout populations following stocking: Insights from an exhaustive study of 72

lakes from Quebec, Canada. Evol Appl. 2014;7: 625–644. doi:10.1111/eva.12160
