# Supplementary material for: Striking Phenotypic Variation yet Low Genetic Differentiation in Sympatric Lake Trout (Salvelinus namaycush)
Source: PLoS One. 2016 Sep 28;11(9):e0162325. doi: 10.1371/journal.pone.0162325 (PMC5040267; doi:10.1371/journal.pone.0162325)
Supplement: S2 File — (PDF) [file pone.0162325.s002.pdf]

Supplemental genetic clustering approaches

To confirm the presence of genetic clustering and differentiation of individuals in our dataset, we employed both non-spatial and spatially-informed analyses. Non-spatial clustering included all individuals (n = 636) whereas a subset of individuals (n = 554) were used for the spatially-informed clustering analyses.

Materials and methods

Non-spatial clustering

In addition to STRUCTURE (see Materials and Methods main paper) we used BAPS (v.6.0) to perform a mixture analysis based on a 'clustering of individuals' model [1]. It was performed for five iterations of each value of K = 1 – 20. We used the maximum likelihood and highest posterior probability to determine the optimal number of K. Subsequently, we performed an admixture analysis [2] on the results of the mixture clustering, using 500 iterations, a minimum of 10 individuals per population, and 100 individuals for each reference individual.

We also used the 'find.clusters' function in a discriminant analysis of principal components (DAPC) using the 'adegenet' package (v.2.0.0) [3] in R (v.3.2.1) [4]. This analysis runs successive K-means clustering and the optimal number of K was selected based on the lowest associated Bayesian Information Criteria (BIC) value after examining the rate of decrease in BIC [5]. We tested values of K = 1 – 20 with five replicates of each K. The DAPC function was executed using this clustering, retaining

axes of principal components analysis (PCA) sufficient to explain 80% of the total variance of the data.

Spatial differentiation

In addition to an sPCA (see Materials and Methods main paper), which incorporated 2-dimensional space (longitude and latitude), we also incorporated depth into our spatial analyses, by performing a 3D sPCA and a distance-based redundancy analysis (dbRDA) in the 'vegan' package (v.2.3-1) [6]. The former was performed using an inverse distance algorithm in which the computed Euclidean distances between locations in 3D space were used to create a graph of neighbouring sites and subsequently a spatial weighting matrix. Depth was also assessed independently using an sPCA. The dbRDA, a constrained ordination technique, was performed including longitude, latitude and depth. Subsequently, we performed partial tests to isolate each variable while controlling for the others, to determine the amount of variance each variable explained.

Results

Non-spatial clustering

Both BAPS and the DAPC model suggest $K > 1$, which is consistent with the results from STRUCTURE; however, there was discordance in the number of K. The BAPS mixture analysis resulted in $K = 9$ as the optimal solution ($\log(ml) = -61011.56$, $P(K=9) = 0.99$); however, there were multiple populations with fewer than 10 individuals and

therefore after an admixture analysis, the true was K = 2 (Fig S2.1). In this analysis, the majority of samples (n = 577) were assigned to a single cluster and few individuals were considered admixed and a total of 15 individuals were removed. Under the DAPC model, BIC values revealed that K = 4 (Fig S2.2). The scatterplot shows that there is substantial overlap between clusters 1 and 2 and are likely admixed; however, clusters 3 and 4 appear to be more genetically distinct.

Overall, the three non-spatial clustering analyses performed all suggest that lake trout of Mistassini Lake exhibit some form of genetic clustering (K > 1). By comparing all three, we can see that STRUCTURE cluster 4 corresponds to BAPS cluster 2 (Fig S2.3; however, the specific clusters identified by the DAPC are inconsistent with both BAPS and STRUCTURE.

Spatial analysis

When a third dimension (depth) was included into the sPCA both as a spatial weighting matrix and independently there were no significant structures identified (nper = 999, Pglobal = 0.285, 0.114 and Plocal = 0.750, 0.430 respectively). When incorporated with longitude and latitude, a similar global pattern was revealed in which individuals were more closely related than expected in the western basin compared to those in the north part of the lake (Fig S2.4). However, when depth is examined independently there is no clear relationship across the lake (Fig S2.4).

The results of the dbRDA suggest that the 3D geographical distribution of lake trout only explains a small (10%) but significant (F = 1.893, P = 0.001) amount of the observed genetic variation. The first two (of three) axes were significant (F = 3.34, 1.57,

both $P < 0.001$ respectively) in explaining this relationship; however, longitude and latitude are collinear (Fig S2.5). Further examination of the geographical variables through the subsequent partial RDAs provide evidence that this relationship was significantly driven by all three variables; however, latitude explained the most variation, followed by depth and longitude to a lesser extent.

Discussion

The above analyses and those described in the main paper provide evidence that lake trout of Mistassini Lake exhibit non-random, biologically significant genetic clustering of individuals with spatial auto-correlation with respect to three dimensional geographic distribution. All non-spatial clustering software suggest K > 1; however, the assignment of these individuals is inconsistent between different analyses. Furthermore, the spatial analyses provide evidence that neighbouring lake trout in certain parts of the lake are more genetically similar to one another than expected by chance, providing evidence for genetic-spatial complexes. Specifically, lake trout found near the mouth of the Rupert River (central west basin) are more closely related to each other compared to those found at the Big Pass (north end of the lake).

References

1. Corander J, Marttinen P, Mantyniemi S. A Bayesian method for identification of stock mixtures from molecular marker data. Fish Bull. 2006;104: 550–558.
2. Corander J, Marttinen P. Bayesian identification of admixture events using multilocus molecular markers. Mol Ecol. 2006;15: 2833–2843. doi:10.1111/j.1365-294X.2006.02994.x
3. Jombart T. adegenet: a R package for the multivariate analysis of genetic markers. Bioinformatics. 2008;24: 1403–1405. doi:10.1093/bioinformatics/btn129
4. Team RC. R: A language and environment for statistical computing. Vienna, Austria: R Foundation for Statistical Computing; 2013.
5. Jombart T, Devillard S, Balloux F. Discriminant analysis of principal components: a new method for the analysis of genetically structured populations. BMC Genet. BioMed Central Ltd; 2010;11: 94. doi:10.1186/1471-2156-11-94
6. Oksanen J, Blanchet FG, Kindt R, Legendre P, Minchin PR, O'Hara R, et al. vegan: Community Ecology Package. R package version 2.3-1 [Internet]. 2015. Available: http://cran.r-project.org/package=vegan

Figures

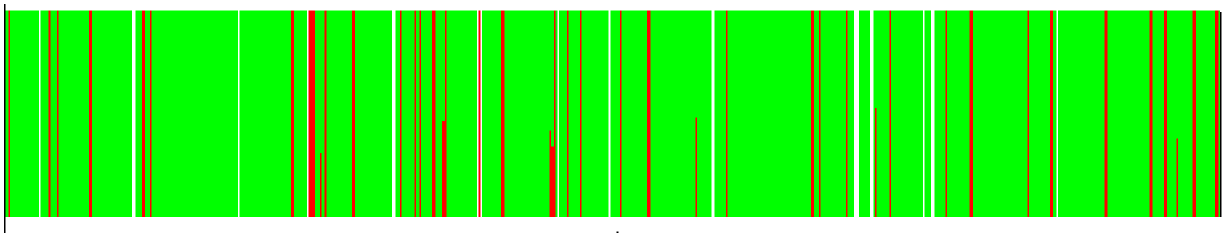

Fig S2.1. Inferred number of clusters (K = 2: green and red) as identified through BAPS. A vertical bar represents an individual's genetic composition assigned to each one of the two clusters. A white bar represents individuals that were removed from the admixture analysis because they were assigned to clusters with less than 10 individuals.

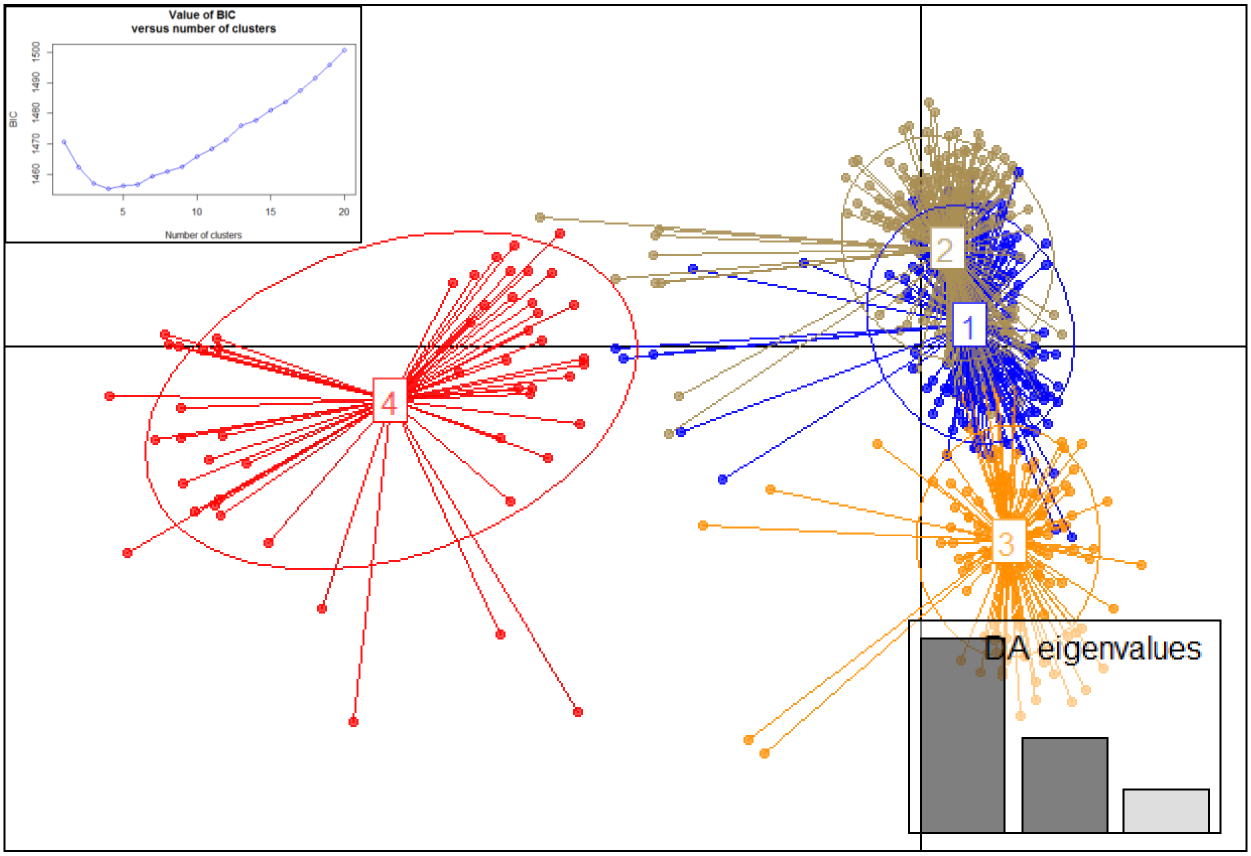

Fig S2.2. **Inference of population structure as identified through DAPC.** Inset are the corresponding BIC plot showing the optimal number of clusters (K = 4) and the DA eigenvalues for the displayed scatterplot.

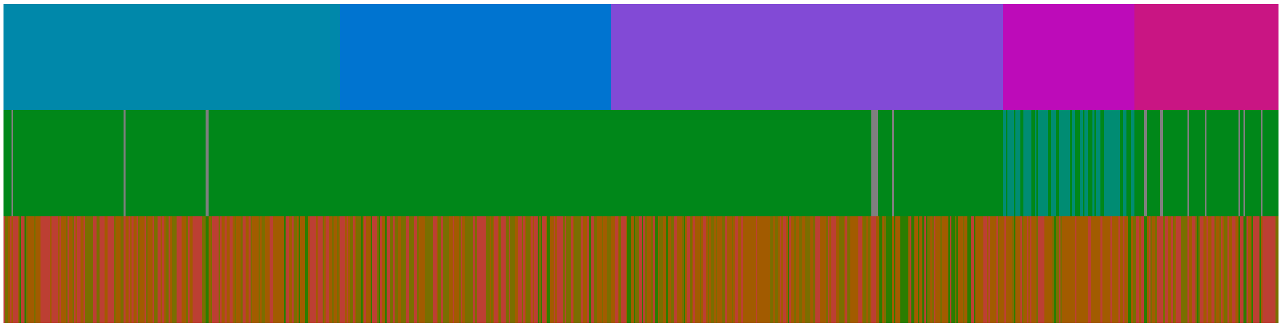

Fig S2.3. Individual assignment of lake trout from the non-spatial clustering analyses. SRUCTURE (K = 5), BAPS (K = 2) and DAPC (K = 4) are shown from top to bottom respectively. It should be noted that the grey vertical bars in BAPS (middle) represent individuals that were excluded from the admixture analysis because they were assigned to clusters with fewer than 10 individuals.

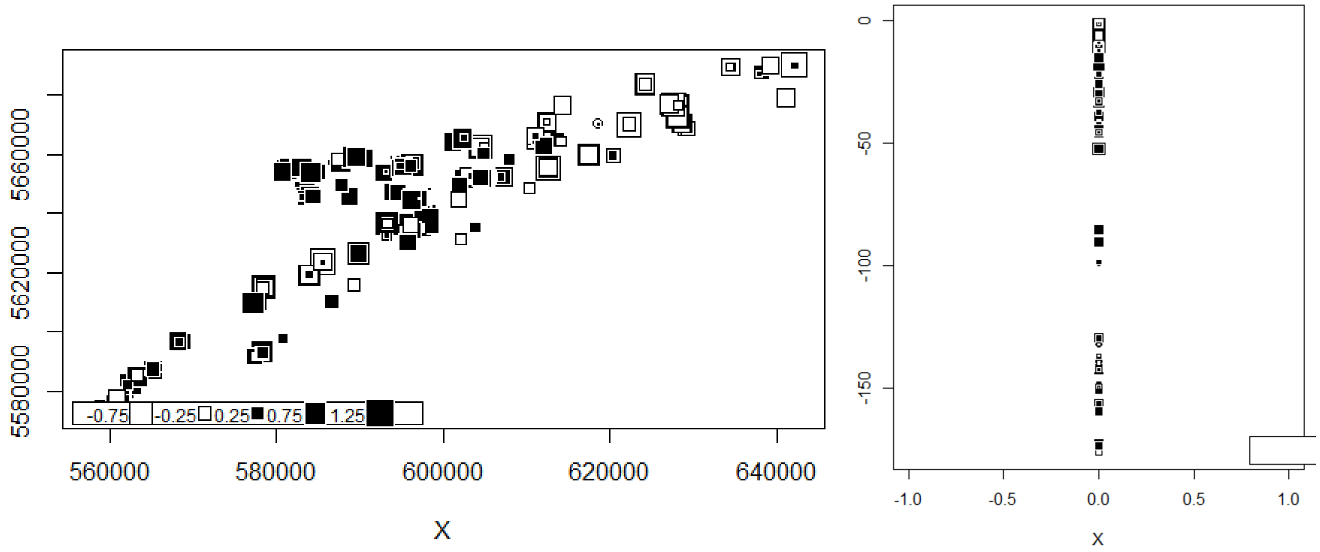

Fig S2.4. The fine scale genetic-geographic complexes present in Mistassini lake trout as revealed by an sPCA. Results show longitude, latitude and depth when analyzed together (left) and when depth is analyzed independently (right). While not significant, the global structures (i.e., neighbouring individuals are more closely related than expected) from both sPCA analyses highlight differences in 3D space. Large black squares are well differentiated from large white squares, but small squares are less differentiated. This shows the spatial-genetic complexes at a finer geographic scale that are present in Mistassini Lake.

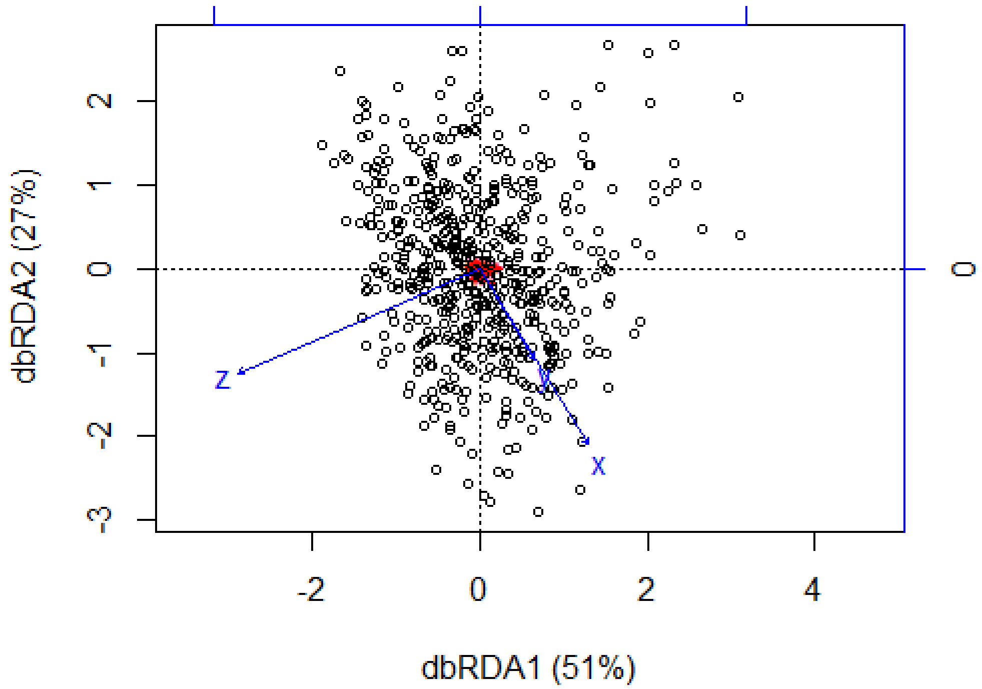

Fig S2.5. Biplot of the distance-based redundancy analysis showing that 3D geographic distances are significant in explaining the observed genetic variation of lake trout in Mistassini Lake. The first two axes (78% of the total variation) show that dbRDA1 is primarily driven by depth (z) and dbRDA2 is primarily driven by latitude (x) and is collinear to longitude (y).
