# Supplementary material for: Striking Phenotypic Variation yet Low Genetic Differentiation in Sympatric Lake Trout (Salvelinus namaycush)
Source: PLoS One. 2016 Sep 28;11(9):e0162325. doi: 10.1371/journal.pone.0162325 (PMC5040267; doi:10.1371/journal.pone.0162325)
Supplement: S3 File — (PDF) [file pone.0162325.s003.pdf]

Genetic differentiation by stepwise mutation (R_{ST})

To investigate whether genetic differentiation may be influenced by stepwise mutations (R_{ST}) we used an allele size randomization procedure (10,000 permutations) in SPAGeDi (v.1.4) [1] across all loci and genetic clusters. If R_{ST} was significantly larger than permuted R_{ST} (pR_{ST}), it suggested that stepwise mutations had an important influence on current differentiation [2].

Allele size permutation tests suggested that stepwise mutations contributed to genetic differentiation: global R_{ST} was significantly greater than pR_{ST} across all clusters (Fig S3.1) and also at five individual loci (Fig S3.2). This mutational influence was most evidenced among five (of 10) cluster comparisons (cluster 3 vs. 1 and 4; 5 vs. 1, 3 vs. 4; Table S3.1).

References

1. Hardy OJ, Vekemans X. SPAGeDi: a verstaile computer program to analyse spatial genetic structure at the individual or population levels. Mol Ecol Notes. 2002;2: 618 – 620.
2. Hardy OJ, Charbonnel N, Fréville H, Heuertz M. Microsatellite allele sizes: a simple test to assess their significance on genetic differentiation. Genetics. 2003;163: 1467–82. Available: http://www.pubmedcentral.nih.gov/articlerender.fcgi?artid=1462522&tool=pmcentrez&rendertype=abstract

Figures & Tables

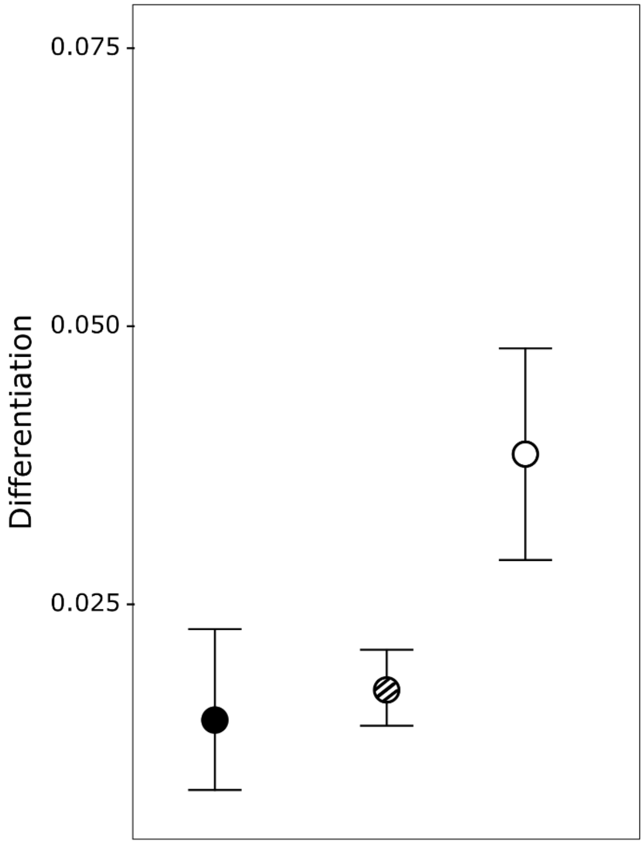

Fig S3.1. Global R_{ST} was significantly greater than pR_{ST} across all clusters. pR_{ST} (black circles), global F_{ST} (hatched circles), R_{ST} (open circles), with their respective 95% confidence intervals at all three thresholds. R_{ST} is significantly greater than pR_{ST} and therefore reveals that the differentiation observed in Mistassini lake trout clusters is due to stepwise mutation and thus indicative of a phylogeographic signal.

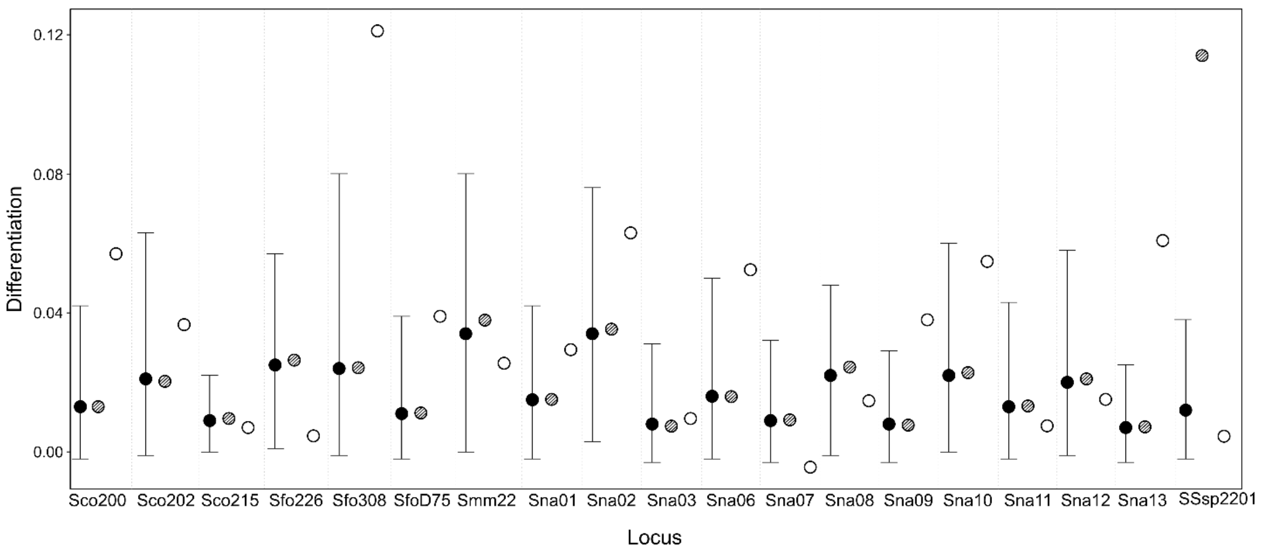

Fig S3.2. R_{ST} was significantly greater than pR_{ST} at five individual loci. pR_{ST} (black circles), F_{ST} (hatched circles), and R_{ST} (open circles) with 95% confidence intervals.

Table S3.1. Results from the allele permutation tests for each cluster pair, across all 19 loci. Under the null hypothesis observed R_{ST} is equal to the mean value after permutation; and under the alternative hypothesis the observed R_{ST} is greater than the mean value after permutation. Therefore, significant fdr corrected *P*-values (bolded) are indicative of a phylogeographic signal.

**Clusters**	*P*-value
1 vs. 2	**0.008**
1 vs. 3	**0.008**
1 vs. 4	**0.000**
1 vs. 5	**0.007**
2 vs. 3	**0.003**
2 vs. 4	0.073
2 vs. 5	**0.004**
3 vs. 4	**0.001**
3 vs. 5	**0.000**
4 vs. 5	**0.019**
