# Supplementary material for: Striking Phenotypic Variation yet Low Genetic Differentiation in Sympatric Lake Trout (Salvelinus namaycush)
Source: PLoS One. 2016 Sep 28;11(9):e0162325. doi: 10.1371/journal.pone.0162325 (PMC5040267; doi:10.1371/journal.pone.0162325)
Supplement: S4 File — (PDF) [file pone.0162325.s004.pdf]

Supplemental morphological figures and tables

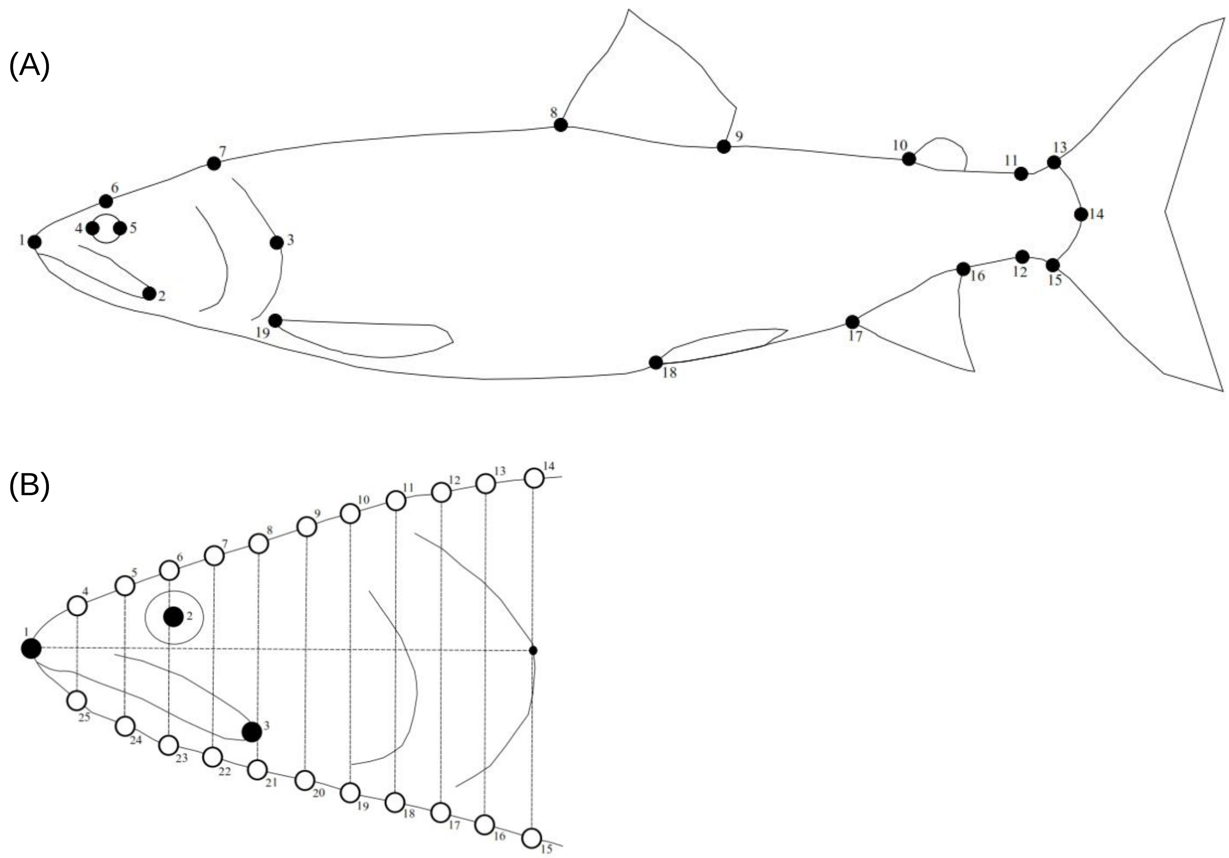

Fig S4.1. Landmarks and semi-landmarks used for morphological analyses of lake trout in Mistassini Lake. (A) Nineteen landmarks used to quantify body shape of lake trout from Mistassini Lake: (1) tip of snout, (2) posterior tip of maxilla, (3) posterior edge of operculum, (4) most anterior part of eye, (5) most posterior part of eye, (6) top of cranium at mid of eye, (7) posterior of cranium above dorsal limit of operculum, (8) anterior insertion of dorsal fin, (9) posterior insertion of dorsal fin, (10) anterior insertion of adipose fin, (11) dorsal position above the narrowest part of caudal peduncle, (12) ventral position below the narrowest part of caudal peduncle, (13) dorsal insertion of caudal fin, (14) mid of the hypural plate, (15) ventral insertion of caudal fin, (16) posterior insertion of anal fin, (17) anterior insertion of anal fin, (18) insertion of pelvic

fin, (19) insertion of pectoral fin. (B) Twenty-two semi-landmarks (white circles, numbers 4-25) and three landmarks (black circles, numbers 1-3) were used to quantify head shape.

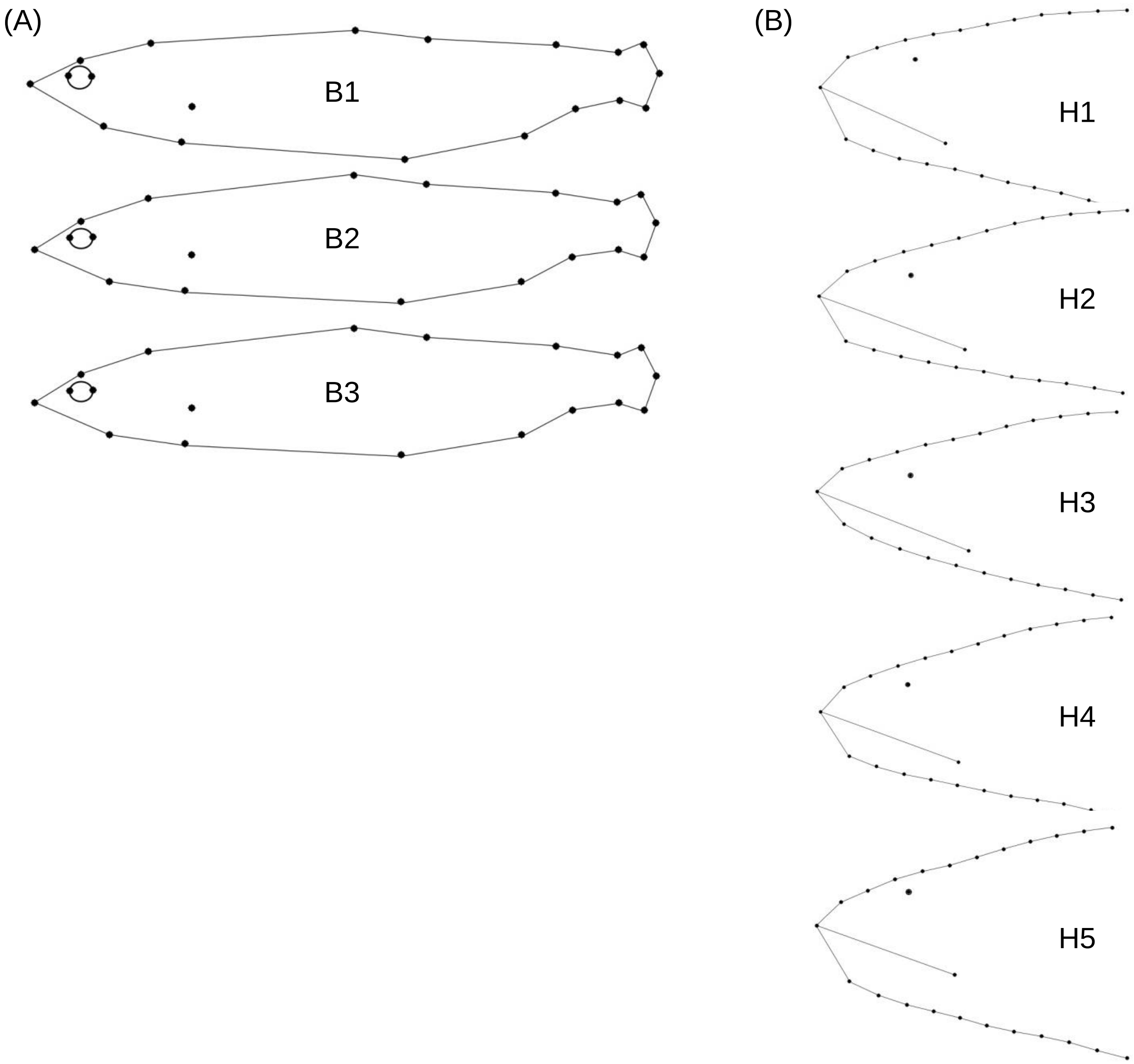

Fig. S4.2. The consensus shape for each one of the identified lake trout morphological clusters detected in Mistassini Lake. Three clusters were identified based on body shape (A: B1 – B3) and five based on head shape (B: H1 – H5).

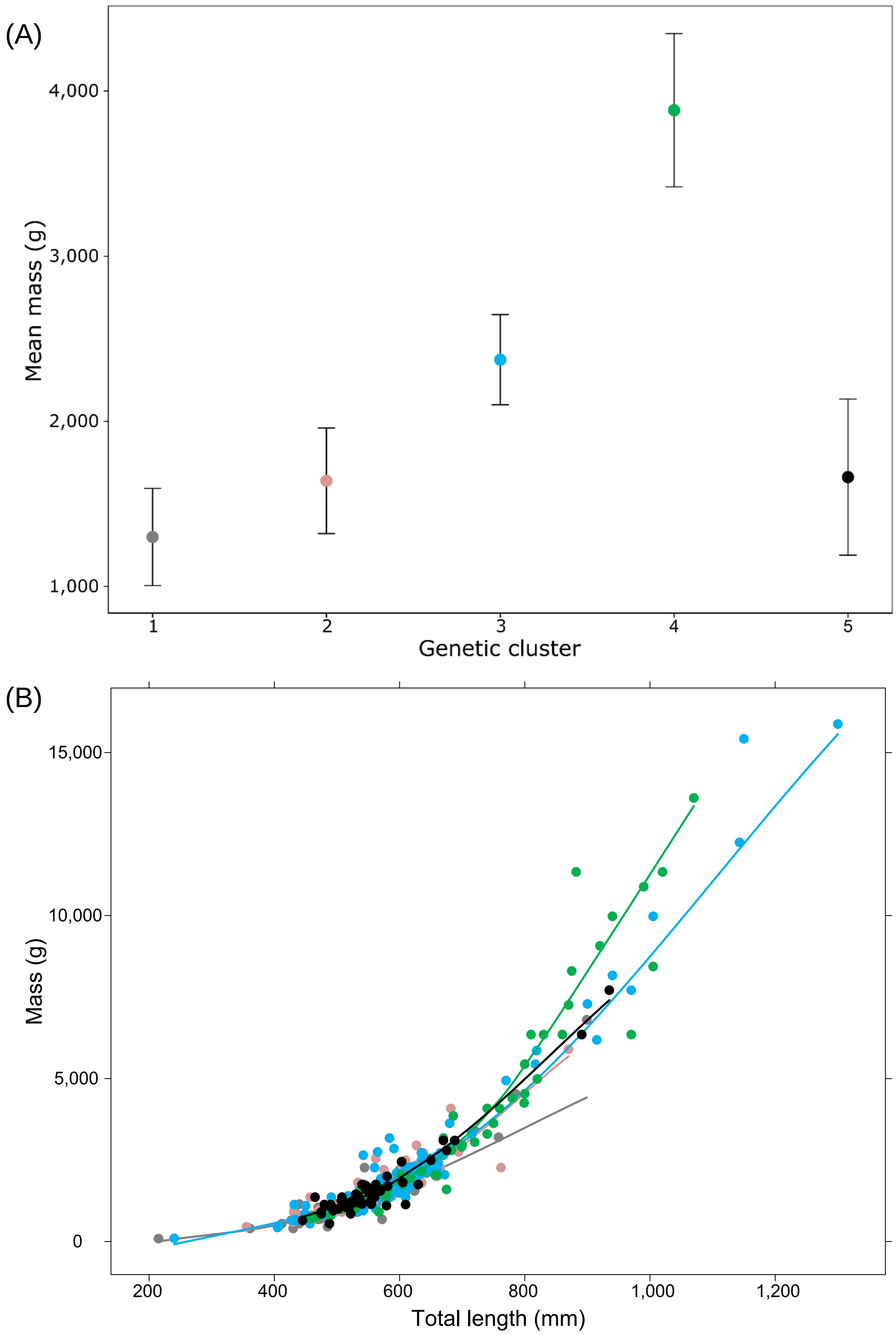

Fig. S4.3. Body size differences between genetically-demarcated lake trout clusters. The mean mass and 95% confidence interval (A) and length/weight curve (B) for each lake trout cluster identified in Mistassini Lake.

Table S4.1. The results of a clustering analysis that was performed in the R package MCLUST. Included are the number of relative warps (RWs), which were produced by previous geometric morphometric analyses of both body and head shape used to assign Mistassini lake trout individuals to morphological clusters. Also reported below is the total percentage of variation explained by these RWs, the type of multivariate clustering model used by MCLUST, the number of clusters, and the mean and uncertainty in group assignments. The number of morphological clusters was determined by selecting the model with the highest Bayesian information criteria (BIC) and is indicated in bold.

RWs used	% variation explained	MCLUST model	No. of clusters	Mean uncertainty ± SE	BIC
		**Body shape**			
2	43	EII	1	0 ± 0	3334
		VII	1		3334
3	55	EII	3	0.22 ± 0.01	5148
		VII	3		5160
4	63	EII	2	0.19 ± 0.01	7044
		VII	3		7064
5	69	EII	3	0.16 ± 0.01	9030
		VII	3		9043
6	74	EII	3	0.14 ± 0.01	11050
		VII	3		11071
7	78	EII	4	0.12 ± 0.01	13095
		**VII**	**3**		**13137**
		**Head Shape**			
2	58	EII	2	0.05 ± 0.01	2402
		VII	1		2396
3	70	**EII**	**5**	**0.17 ± 0.01**	**3811**
		VII	2		3805

Table S4.2. Confusion matrix results for jackknifed discriminant function analysis for body (A) and head (B) morphological clusters.

(A)

	Predicted Cluster			
**Given cluster**	**1**	**2**	**3**	**Total**
**1**	132	5	7	144
**2**	3	105	3	111
**3**	4	2	20	26
**Total**	139	112	30	281

(B)

	Predicted Cluster					
**Given cluster**	**1**	**2**	**3**	**4**	**5**	**Total**
**1**	19	0	0	0	0	19
**2**	1	42	2	0	0	45
**3**	0	0	22	0	0	22
**4**	8	14	10	137	8	177
**5**	0	0	0	0	18	18
**Total**	28	56	34	137	26	281
