# Supplementary material for: Striking Phenotypic Variation yet Low Genetic Differentiation in Sympatric Lake Trout (Salvelinus namaycush)
Source: PLoS One. 2016 Sep 28;11(9):e0162325. doi: 10.1371/journal.pone.0162325 (PMC5040267; doi:10.1371/journal.pone.0162325)
Supplement: S5 File — (PDF) [file pone.0162325.s005.pdf]

Multinomial regression analysis

To confirm the association between genetic clusters and ecological associations, we performed a multinomial regression analysis in the 'nnet' package (v.7.3-9) [1] in R [2] in which individuals were weighted based on their highest *q* value. Model selection was based on Akaike's information criterion (AIC) [3].

The best fit model using multinomial regression included both (water) depth and sector as the main influences on genetic clustering (Table S5.1; McFadden's pseudo-R^2 value = 0.45). Cluster 1 and 4 individuals were disproportionately caught in deeper water and mid-depths respectively, whereas clusters 2, 3 and 5 typically occupied shallow water (Fig 1C, Table S5.2). Cluster 1 was captured predominantly in sectors E9, W3 and W4, clusters 2 and 3 in sector W3 and W4, cluster 4 in sector W3, and cluster 5 in sector E11. However, individuals from each cluster were captured in almost every sector except E12 and E7 wherein only one individual per sector was captured.

References

1. Venables W, Ripley B. Modern Applied Statistics with S. New York: Springer; 2002.
2. Team RC. R: A language and environment for statistical computing. Vienna, Austria: R Foundation for Statistical Computing; 2013.
3. Akaike H. A new look at the statistical model identification. IEEE Trans Autom Control. 1974;19: 716–723. doi:10.1109/TAC.1974.1100705

Tables

Table S5.1: Model selection results for the multinomial regression analysis based on the Akaike information criterion (AIC). The model with the lowest AIC value (and the most parsimonious) was selected (bold).

Model	AIC
**Pop ~ log(Depth) + Sector**	**1159.173**
Pop ~ log(Depth) + (Basin/Sector)	1159.173
Pop ~ log(Depth) + Basin + Sector	1159.173
Pop ~ log(Depth) + Basin	1177.086
Pop ~ log(Depth) + log(Depth):Basin	1178.213

Table S5.2: Coefficients of the multinomial regression analysis in which cluster 1 was the reference. Depth (m) was logged and sectors (W1 – W6 and E8 – E11) were the only two ecological variables carried forward from previous model selection.

**Cluster**	**Log (depth)**	**E8**	**E9**	**E10**	**E11**	**W1**	**W2**	**W3**	**W4**	**W5**	**W6**
2	-1.7	-2.6	-2.1	6.1	-1.8	11.3	-0.6	-1.8	-0.9	-0.5	-28.2
3	-2.0	-1.4	-1.7	6.5	-1.9	12.2	-0.1	-1.7	0.2	0.4	-1.1
4	-0.7	14.0	-11.8	-13.1	13.3	27.6	15.1	14.9	14.5	15.8	15.8
5	-1.4	-0.7	-2.1	4.2	0.7	12.6	-26.2	-2.2	-0.6	-0.2	0.0
