# Supplementary material for: Striking Phenotypic Variation yet Low Genetic Differentiation in Sympatric Lake Trout (Salvelinus namaycush)
Source: PLoS One. 2016 Sep 28;11(9):e0162325. doi: 10.1371/journal.pone.0162325 (PMC5040267; doi:10.1371/journal.pone.0162325)
Supplement: S6 File — (PDF) [file pone.0162325.s006.pdf]

Supplemental genetic data of lake trout in Mistassini Lake

Table S6.1. Basic descriptive statistics for 19 loci across all 5 genetically-differentiated clusters as identified by STRUCTURE. Reported are the average number of alleles per locus (N_A), observed (H_O) and expected (H_E) heterozygosity, and allelic richness (A_R; based on 584 individuals).

	Sco202	Sfo226	Sfo308	SfoD75	Sna02	Sna03	Sna06	Sna07	Sna08	Sna09	Sna10	Sco200	Sco215	Sna11	Smm22	Sna01	Sna12	Sna13	SSsp2201	Avg.
**Cluster 1 (n=168)**																				
N_A	13	29	26	13	13	29	36	21	12	55	26	32	12	27	16	23	22	21	19	23.42
H_E	0.83	0.86	0.92	0.86	0.82	0.89	0.93	0.93	0.49	0.96	0.89	0.94	0.56	0.85	0.67	0.90	0.88	0.88	0.90	0.84
H_O	0.83	0.82	0.95	0.86	0.82	0.90	0.96	0.84	0.49	0.92	0.90	0.96	0.45	0.85	0.66	0.89	0.85	0.85	0.87	0.82
A_R	12.38	23.58	20.79	11.79	10.05	23.74	27.33	19.23	9.71	40.43	21.05	26.86	8.98	20.45	77.88	19.11	18.16	17.61	15.80	18.89
**Cluster 2 (n=135)**																				
N_A	12	24	25	12	12	26	35	22	15	47	22	31	12	22	15	20	19	22	17	21.58
H_E	0.80	0.83	0.92	0.86	0.79	0.87	0.94	0.92	0.67	0.97	0.81	0.93	0.48	0.80	0.81	0.89	0.86	0.89	0.90	0.84
H_O	0.78	0.79	0.91	0.84	0.73	0.86	0.88	0.82	0.64	0.97	0.85	0.91	0.45	0.82	0.81	0.88	0.83	0.85	0.87	0.82
A_R	10.40	20.68	21.48	10.93	11.53	20.51	28.68	18.84	12.95	40.65	19.26	26.27	8.82	17.68	12.63	17.35	16.32	18.96	15.98	18.42
**Cluster 3 (n=195)**																				
N_A	13	27	25	14	13	30	43	27	15	61	25	30	11	26	16	17	22	22	21	24.11
H_E	0.84	0.72	0.91	0.85	0.76	0.88	0.95	0.92	0.68	0.97	0.92	0.93	0.48	0.82	0.79	0.87	0.92	0.90	0.88	0.84
H_O	0.81	0.72	0.93	0.87	0.72	0.85	0.92	0.81	0.69	0.96	0.90	0.93	0.48	0.78	0.78	0.84	0.88	0.85	0.86	0.82
A_R	12.08	20.09	19.66	10.94	10.38	21.47	31.75	21.72	12.24	45.85	19.89	24.48	7.92	20.29	13.31	14.16	19.25	19.37	16.87	19.04
**Cluster 4 (n=66)**																				
N_A	11	14	18	10	7	24	28	17	6	33	15	21	7	16	12	17	14	13	13	15.58
H_E	0.81	0.73	0.89	0.86	0.63	0.88	0.93	0.89	0.35	0.95	0.90	0.91	0.62	0.84	0.71	0.89	0.87	0.85	0.80	0.81
H_O	0.83	0.65	0.88	0.80	0.65	0.80	0.92	0.81	0.36	0.85	0.85	0.95	0.68	0.83	0.78	0.97	0.85	0.86	0.86	0.80
A_R	10.99	13.64	17.81	9.94	6.88	23.75	27.38	17.00	5.94	32.38	14.95	20.84	6.91	15.58	11.95	17.00	13.88	12.94	12.81	15.40
**Cluster 5 (n=72)**																				
N_A	11	19	23	9	12	19	28	19	11	39	18	23	9	16	11	16	18	18	15	17.58
H_E	0.80	0.89	0.91	0.83	0.83	0.85	0.91	0.92	0.49	0.95	0.89	0.93	0.52	0.78	0.70	0.87	0.85	0.85	0.88	0.82
H_O	0.81	0.83	0.92	0.85	0.79	0.77	0.93	0.86	0.54	0.92	0.90	0.94	0.57	0.75	0.65	0.81	0.76	0.82	0.89	0.80
A_R	10.56	18.44	22.33	9.00	11.56	18.27	26.30	18.87	10.44	37.36	17.49	22.46	8.70	15.36	10.44	15.77	17.54	17.63	14.30	16.99
**Global**																				
N_A	13	35	32	15	15	35	60	31	18	72	30	38	17	37	18	24	25	24	22	29.53
H_E	0.84	0.82	0.93	0.87	0.80	0.89	0.95	0.93	0.59	0.97	0.90	0.94	0.53	0.83	0.77	0.90	0.90	0.89	0.89	0.85
H_O	0.81	0.76	0.92	0.85	0.75	0.84	0.92	0.83	0.55	0.92	0.88	0.94	0.53	0.81	0.74	0.88	0.84	0.84	0.87	0.81
A_R	13.00	34.75	31.83	14.99	14.92	34.93	59.55	30.83	17.91	71.22	29.73	37.67	16.59	36.27	17.84	24.00	24.84	24.00	21.93	29.30

Table S6.2. Estimation of exact *P*-values by Markov chain method for Hardy-Weinberg heterozygote deficiencies for all 19 loci. Shown are the results for the null hypothesis of no population structuring and alternative hypothesis of five clusters as identified by STRUCTURE. All analyses were carried out using GENEPOP. Bold *P*-values indicate significance (*P* < 0.05). Under the alternative hypothesis *P*-values have been adjusted using the fdr method for 95 comparisons.

Cluster	n	Sco202	Sfo226	Sfo308	SfoD75	Sna02	Sna03	Sna06	Sna07	Sna08	Sna09	Sna10	Sco200	Sco215	Sna11	Smm22	Sna01	Sna12	Sna13	SSsp2201
**Null hypothesis (K = 1)**																				
**1**	636	**0.001**	**0.000**	0.171	**0.047**	**0.000**	**0.000**	**0.025**	**0.000**	0.351	**0.000**	0.091	0.078	**0.000**	**0.002**	**0.049**	**0.034**	**0.000**	**0.002**	**0.001**
**Alternative hypothesis (K = 5)**																				
**1**	168	0.265	0.365	0.989	0.388	0.614	0.312	1.000	**0.000**	0.255	0.072	0.275	0.669	**0.000**	0.274	0.175	0.090	0.170	0.058	0.255
**2**	135	**0.034**	**0.000**	0.345	0.198	**0.016**	0.240	0.199	**0.002**	0.435	0.275	0.989	0.221	0.388	0.526	0.388	0.665	0.510	0.059	0.312
**3**	195	0.243	**0.003**	0.577	0.873	0.243	0.058	**0.000**	**0.000**	0.914	0.526	0.114	0.388	**0.012**	0.265	0.299	0.175	0.265	0.198	0.337
**4**	66	0.105	0.136	0.289	0.170	0.812	**0.044**	0.243	**0.005**	0.899	**0.000**	0.148	0.970	0.970	0.669	0.881	0.996	0.307	0.614	0.614
**5**	72	0.436	**0.000**	0.312	0.302	**0.000**	0.067	0.538	**0.013**	0.812	0.175	0.899	0.607	0.996	0.265	0.526	0.436	**0.000**	0.206	0.148

Table S6.3. Linkage disequilibrium results for all 19 loci of Mistassini lake trout.

Shown are the number of significant locus pairs for all 19 loci under the null hypothesis of no population structuring and the alternative hypothesis of five clusters as identified by STRUCTURE.

**Cluster**	**n**	**Level of significance**	
		Uncorrected *P*-value	FDR corrected *P*-value
**Null hypothesis***			
1	636	20	7
**Alternative hypothesis†**			
1	168	3	0
2	135	15	3
3	195	10	2
4	66	34	0
5	72	36	10
Across all clusters		23	12

*Under the null hypothesis there were a total of 171 comparisons.

†Under the alternative hypothesis there were a total of 855 comparisons.

Table S6.4. Statistical results from STRUCTURE (20 iterations) for 636 individuals across 19 loci. Shown are the mean and standard deviations (SD) of the log-likelihood values of the data (LnP[D]) for a given number of clusters (K). In addition, presented is the mean value of ΔK, the ad hoc statistic of Evanno et al. 2005, used to summarize the second-order rate of change in LnP[D]. Both test statistics (bolded) indicate that the most likely number of genetic clusters is 5.

**K**	**meanLnP[D]**	**SDLnP[D]**	**ΔK**
1	-59327.66	0.51	NA
2	-58945.29	5.24	29.46
3	-58717.37	7.49	10.01
4	-58414.33	5.24	9.70
5	**-58060.42**	8.37	**57.03**
6	-58184.06	92.73	11.00
7	-59328.24	1023.45	0.87
8	-59578.83	966.59	0.83
9	-60637.82	1127.27	1.38
10	-60132.61	1132.78	0.28

Table S6.5. Pairwise F_{ST} for genetic clusters as identified by STRUCTURE and morphological clusters (body and head) as identified by MCLUST. Values that differed significantly from zero are bolded.

	F_{ST}
**Genetic clusters**	
1 vs. 2	**0.0143**
1 vs. 3	**0.0134**
1 vs. 4	**0.0206**
1 vs. 5	**0.0143**
2 vs. 3	**0.0118**
2 vs. 4	**0.0283**
2 vs. 5	**0.0204**
3 vs. 4	**0.0249**
3 vs. 5	**0.0197**
4 vs. 5	**0.0355**
**Morphological clusters: body**	
B1 vs. B2	0.0001
B1 vs. B3	0.0023
B2 vs. B3	0.0009
**Morphological clusters: head**	
H1 vs. H2	0.0006
H1 vs. H3	0.0025
H1 vs. H4	-0.0021
H1 vs. H5	-0.0002
H2 vs. H3	0.0003
H2 vs. H4	0.0001
H2 vs. H5	-0.0004
H3 vs. H4	0.0012
H3 vs. H5	0.0005
H4 vs. H5	0.0004
