# Supplementary material for: Striking Phenotypic Variation yet Low Genetic Differentiation in Sympatric Lake Trout (Salvelinus namaycush)
Source: PLoS One. 2016 Sep 28;11(9):e0162325. doi: 10.1371/journal.pone.0162325 (PMC5040267; doi:10.1371/journal.pone.0162325)
Supplement: S7 File — Effective population size (Ne) and gene flow Mistassini lake trout. (PDF) [file pone.0162325.s007.pdf]

Effective population size (N_e) and gene flow Mistassini lake trout

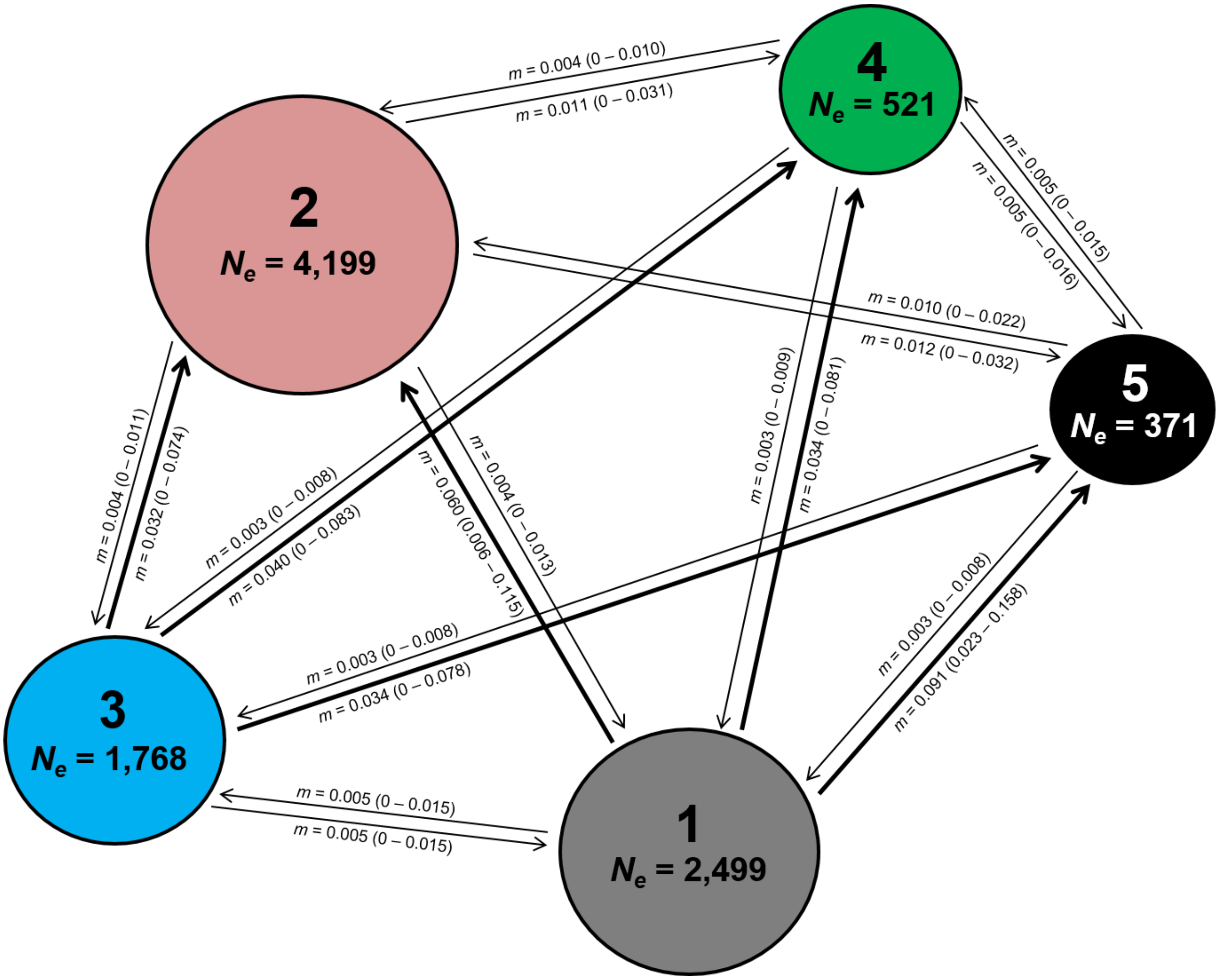

Fig S7.1: Schematic summary showing inferred contemporary gene flow (*m*). It is the proportion of the genetic cluster that migrated per generation in the direction shown by the arrow) with 95% confidence intervals (in parentheses) between lake trout clusters present in Mistassini Lake. The size of each cluster circle corresponds to the point estimates of the effective population size (N_e; see Table S7.1 (below) for 95% confidence intervals). Estimates of recent gene flow were based on all individuals (n = 636) and results were averaged over five iterations using BayesAss.

Table S7.1. Effective population size (*N_e*) estimates and associated 95% confidence interval (CI) for each genetically-demarcated cluster identified in Mistassini Lake. All 636 samples and their respective sample sizes per population (n) were used for this analysis. Critical values (P_{crit}), allele frequencies less than 0.01 were used for larger (> 100 individuals) populations and 0.02 was used for smaller (< 100 individuals) populations.

Cluster	n	N_e	95% CI
1	168	2499	1471 – 7888
2	135	4199	1744 – ∞
3	195	1768	1246 – 2997
4	66	521	328 – 1209
5	72	371	274 – 566

Table S7.2. The time required for FST to reach a new equilibrium. The genetic differentiation (F_{ST}), mean gene flow into each cluster (recent migration rates, *m*), mean effective population size (N_e), the time, in generations, required for F_{ST} to reach halfway ($t_{1/2}$) and completely (~ t) to a new equilibrium for each cluster pair and globally, based on all individuals (n = 636).

Cluster pair	F_{ST}	mean *m*	mean N_e	$t_{1/2}$	~ t
**1 – 2**	0.0143	0.0151	3349	22.69	46
**1 – 3**	0.0134	0.0038	2134	88.31	177
**1 – 4**	0.0206	0.0133	1510	25.64	45
**1 – 5**	0.0143	0.0197	1435	17.31	35
**2 – 3**	0.0118	0.015	2984	22.82	35
**2 – 4**	0.0283	0.0245	2360	13.94	22
**2 – 5**	0.0204	0.0308	2285	11.03	51
**3 – 4**	0.0249	0.0132	1145	25.73	51
**3 – 5**	0.0197	0.0196	1070	17.35	28
**4 – 5**	0.0355	0.029	446	11.55	23
**Global**	0.017	0.0184	1872	18.55	37
