# Supplementary material for: Striking Phenotypic Variation yet Low Genetic Differentiation in Sympatric Lake Trout (Salvelinus namaycush)
Source: PLoS One. 2016 Sep 28;11(9):e0162325. doi: 10.1371/journal.pone.0162325 (PMC5040267; doi:10.1371/journal.pone.0162325)
Supplement: S8 File — (PDF) [file pone.0162325.s008.pdf]

Supplemental tables and figures for associations between genetic, morphological and ecological differentiation

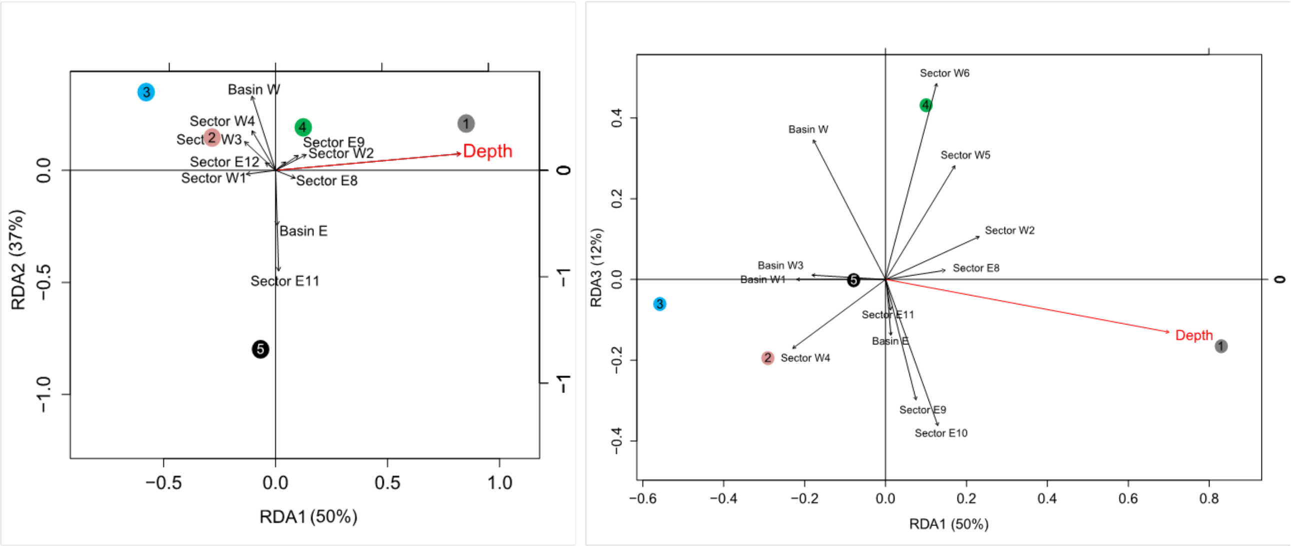

Fig S8.1. Results of redundancy analysis (RDA) for the first three axes (99% of variation). These results demonstrate that depth is significant with respect to the observed genetic population structuring. Coloured circles represent the 5 genetically-distinct lake trout clusters present in Mistassini Lake. Ecological variables (depth, basin and sector) and their associated arrows represent the nature of the relationship with respect to each cluster.

(A)

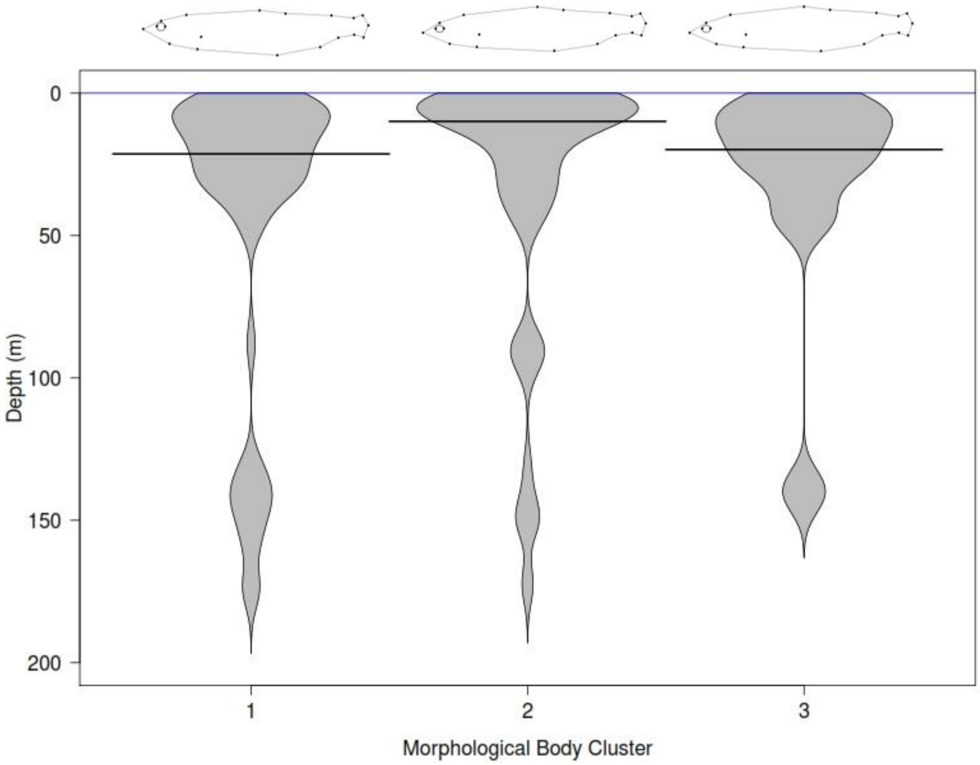

(B)

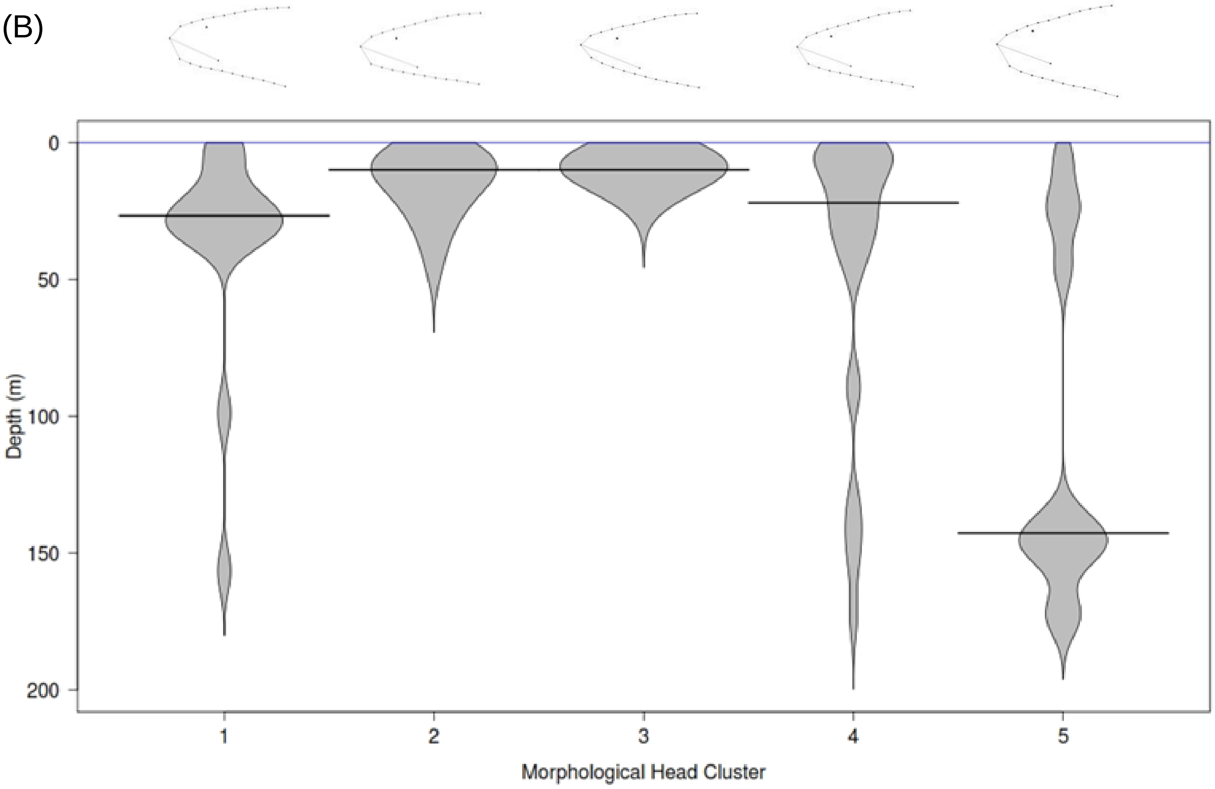

Fig S8.2. Beanplot showing the depth variation for each lake trout morphological

cluster based on body (A) and head (B) shape. The black line represents the median and inset images represent the consensus shape for morphological cluster identified in Mistassini Lake.

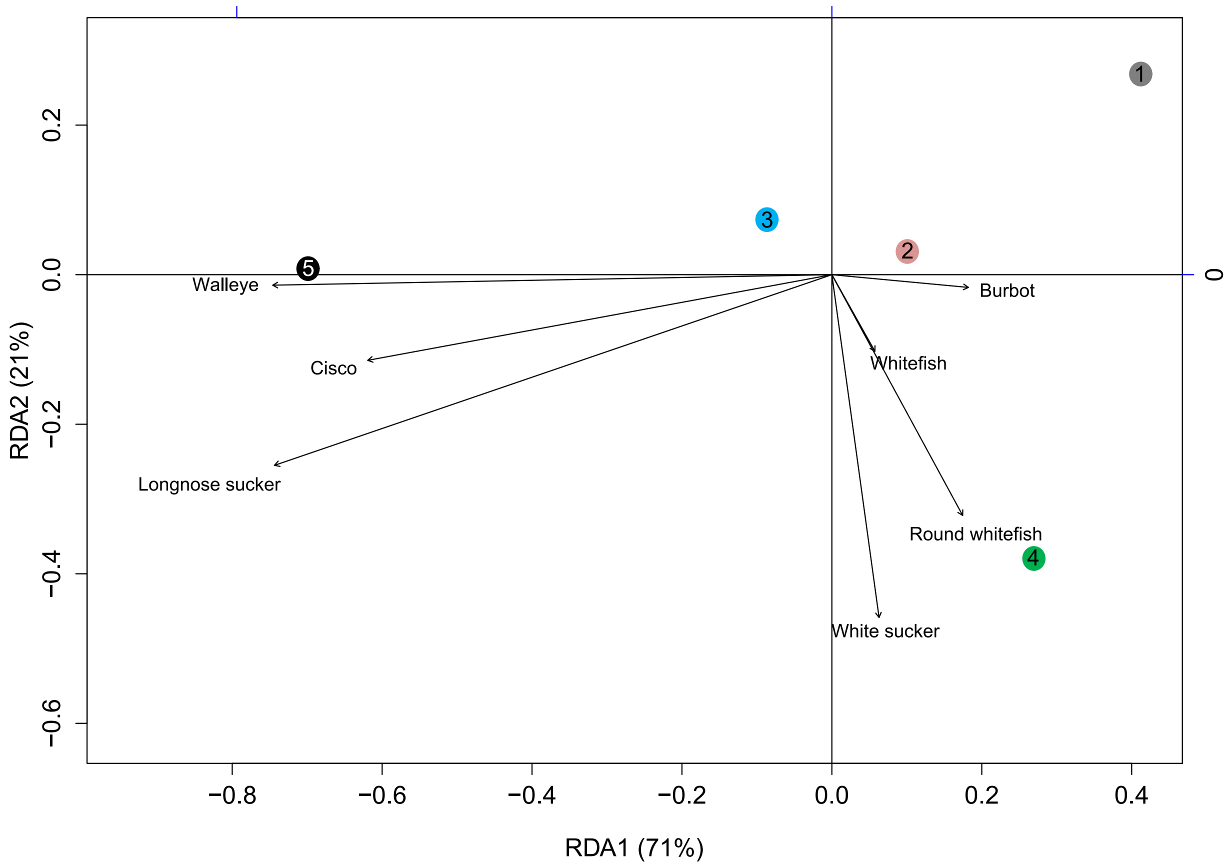

Fig S8.3. Visualization of bycatch species association with each lake trout cluster (coloured circle) identified in Mistassini Lake.

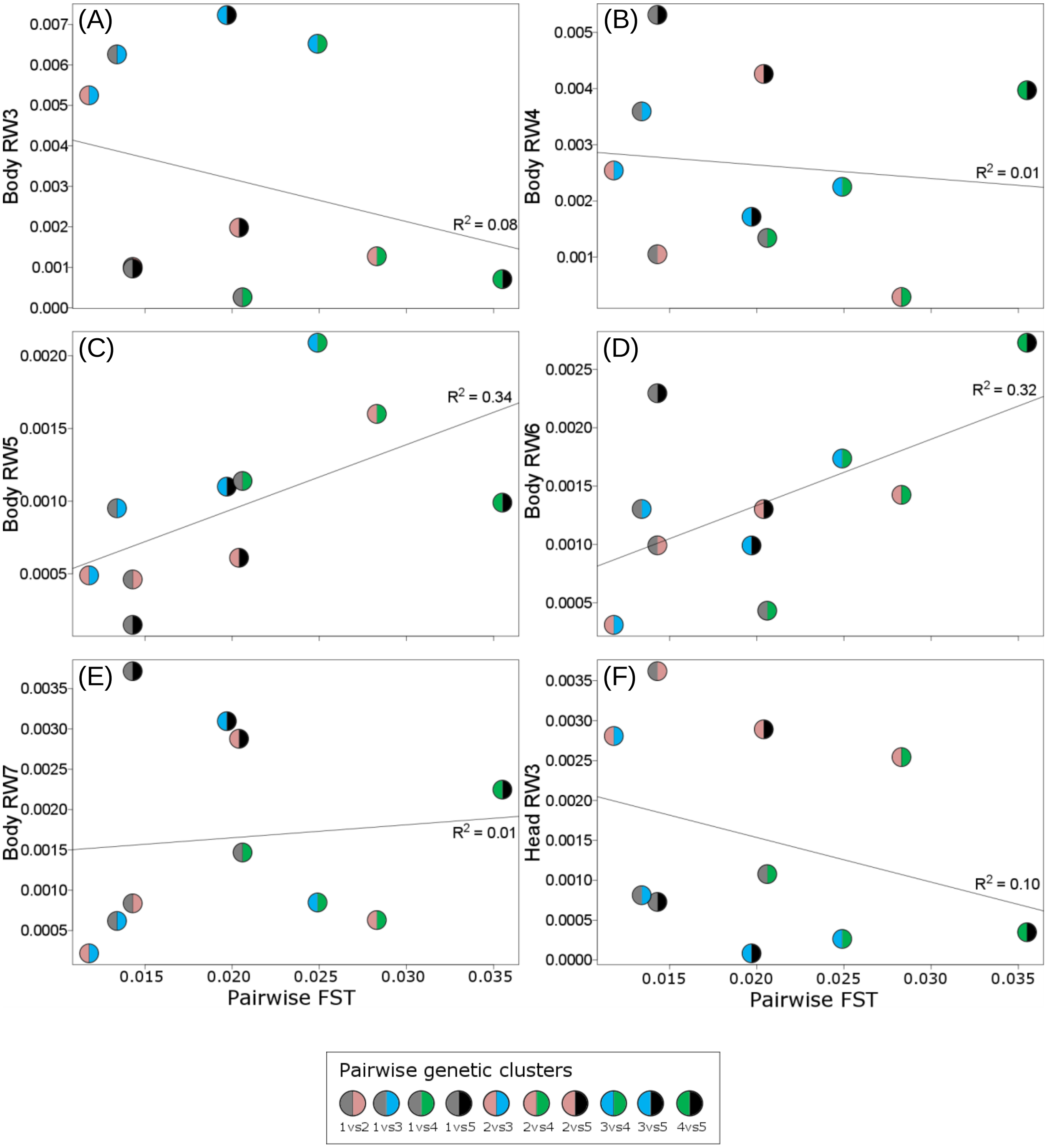
(A)
Body RW3
$R^2 = 0.08$
(B)
Body RW4
$R^2 = 0.01$
(C)
Body RW5
$R^2 = 0.34$
(D)
Body RW6
$R^2 = 0.32$
(E)
Body RW7
$R^2 = 0.01$
(F)
Head RW3
$R^2 = 0.10$
Pairwise FST
Pairwise FST
Pairwise genetic clusters
1vs2 1vs3 1vs4 1vs5 2vs3 2vs4 2vs5 3vs4 3vs5 4vs5

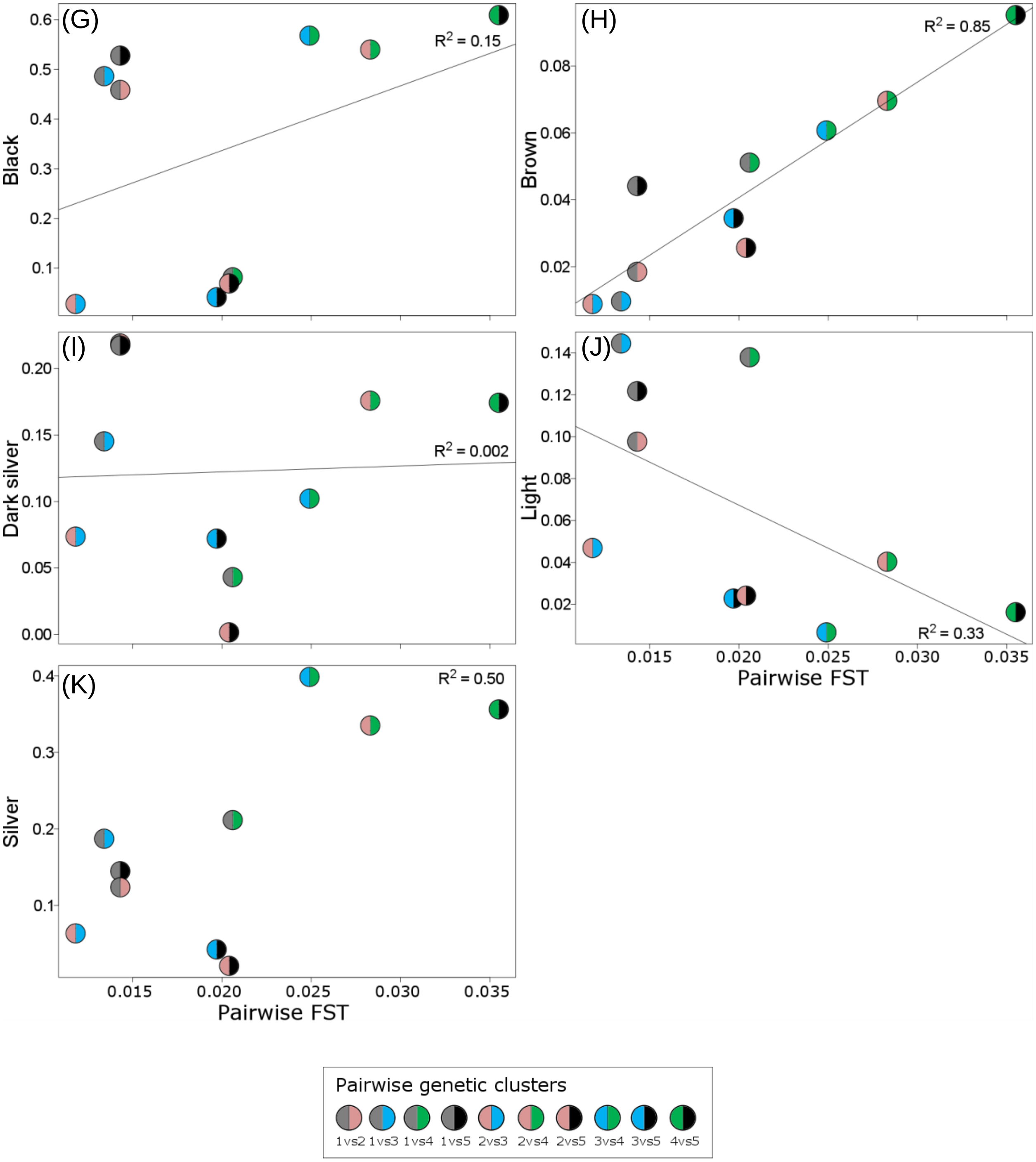

Fig. S8.4. Visualisations of the remaining Mantel tests between genetic distance and morphological and ecological variables. Shown is the genetic distance (pairwise F_{ST}) vs.(i) the absolute difference in mean relative warp (RW) score for RW3 – 7 for body (A

– E); (ii) RW3 for body morphology (F); and the absolute difference in colour frequencies (G – K).

Table 8.1. Results from Mantel tests between genetic distance and morphological and ecological variables. Individual tests were between the genetic distance (pairwise F_{ST}) and (i) the absolute difference in mean relative warp (RW) score for the first seven RWs for body and first three RWs for head morphology; (ii) the absolute difference in prey abundance; (iii) the absolute difference in median depth (m); and (iv) difference in observed colour frequencies. Bolded *P*-values indicate significance. The Mantel R statistic is based on Pearson's product-moment correlation.

Pairwise F_{ST} vs.	*P*-value	R
Body RW1	0.142	0.629
Body RW2	0.233	0.544
Body RW3	0.642	-0.282
Body RW4	0.600	-0.113
Body RW5	0.158	0.580
Body RW6	0.083	0.566
Body RW7	0.383	0.098
Head RW1	0.250	0.342
Head RW2	0.108	0.772
Head RW3	0.667	-0.322
Bycatch	0.367	0.128
Depth	0.400	0.098
Black	**0.017**	0.392
Brown	**0.008**	0.927
Dark silver	0.450	0.044
Light	0.958	-0.575
Silver	0.092	0.708
