# Supplementary material for: Striking Phenotypic Variation yet Low Genetic Differentiation in Sympatric Lake Trout (Salvelinus namaycush)
Source: PLoS One. 2016 Sep 28;11(9):e0162325. doi: 10.1371/journal.pone.0162325 (PMC5040267; doi:10.1371/journal.pone.0162325)
Supplement: S9 File — (PDF) [file pone.0162325.s009.pdf]

Assessment of recent bottlenecks of Mistassini lake trout

As post-glacial colonization can be associated with bottlenecks, we also tested for bottlenecks in each demarcated genetic cluster using BOTTLENECK (v.1.2.02) [1]. This program compares heterozygosity excess (H_E) to that expected (H_{EQ}) under mutation-drift equilibrium. During bottlenecks allelic richness is assumed to decline more rapidly than heterozygosity, therefore, populations that have undergone recent bottlenecks should reveal a greater H_E to H_{EQ} ratio [2]. This test was computed under both the step-wise mutation (SMM) and two-phase mutation (TPM) models using 1000 iterations, and default settings (variance for the geometric distribution for TMP = 30 and proportion of SMM in TPM = 70% as recommended by Piry et al. [2]); significance was determined using Wilcoxon signed rank tests ($P < 0.05$). There was no evidence of recent bottlenecks in any demarcated populations under either mutation model employed (Table S9.1).

References

1. Cornuet JM, Luikart G. Description and power analysis of two tests for detecting recent population bottlenecks from allele frequency data. Genetics. 1996;144: 2001–2014.
2. Piry S, Luikart G, Cornuet JM. BOTTLENECK: A computer program for detecting recent reductions in the effective population size using allele frequency data. J Hered. 1999;90: 502–503. doi:10.1093/jhered/90.4.502

Table S9.1. Results for the detection of any recent bottlenecks across all loci and genetically-demarcated clusters in Mistassini Lake. Shown are the results from the stepwise (SMM) and two-phase models (TMP) of mutation. The number of loci with heterozygosity excess (H_E) and expected number of loci with heterozygosity excess (H_{EQ}) are reported. Statistical significance of deviation from either model is represented by *P*, which is a one tail Wilcoxon sign rank test for heterozygosity excess across 19 loci for each cluster.

	TPM			SMM		
**Cluster**	H_E	H_{EQ}	*P*	H_E	H_{EQ}	*P*
**1**	7	11.21	0.82	2	11.29	0.99
**2**	8	11.22	0.89	2	11.18	1.00
**3**	8	11.18	0.75	3	11.34	0.99
**4**	6	11.13	0.86	5	11.24	0.99
**5**	8	11.26	0.79	4	11.34	0.99
